# Unveiling the dynamic trends of plant-derived exosome nanovesicles-based theranostics: through bibliometric and visualized analysis

**DOI:** 10.3389/fmed.2025.1553915

**Published:** 2025-06-24

**Authors:** Siyang Cao, Yingchen Pang, Yihao Wei, Deli Wang, Ao Xiong, Jun Yang, Hui Zeng

**Affiliations:** ^1^Department of Bone & Joint Surgery, Peking University Shenzhen Hospital, Shenzhen, Shenzhen, Guangdong, China; ^2^Shenzhen Key Laboratory of Orthopaedic Diseases and Biomaterials Research, Peking University Shenzhen Hospita, Shenzhen, Guangdong, China; ^3^Department of Pulmonary and Critical Care Medicine, Peking University Shenzhen Hospital, Shenzhen, Guangdong, China; ^4^Department of Pulmonary and Critical Care Medicine, Shenzhen Xinhua Hospital, Shenzhen, Guangdong, China; ^5^Department of Rehabilitation Science, The Hong Kong Polytechnic University, Hong Kong Special Administrative Region, Hong Kong, China; ^6^Faculty of Pharmaceutical Sciences, Shenzhen Institute of Advanced Technology, Chinese Academy of Sciences (CAS), Shenzhen, Guangdong, China; ^7^Faculty of Pharmaceutical Sciences, Shenzhen University of Advanced Technology, Shenzhen, Guangdong, China; ^8^Department of Radiology, Peking University Shenzhen Hospital, Shenzhen, Guangdong, China; ^9^Department of Orthopedics, Shenzhen Second People’s Hospital, The First Affiliated Hospital of Shenzhen University, Shenzhen, Guangdong, China

**Keywords:** plant-derived exosome-like nanoparticles, biotherapeutic applications, delivery strategy, bibliometrics, visualized analysis, theranostics

## Abstract

Plant-derived exosome nanovesicles (PDENs) have emerged as eco-friendly, sustainable and highly efficient platforms for drug delivery, attracting significant attention in biomedical research. As a consequence, PDENs have become a focus for multidisciplinary investigation. Despite extensive research, impartial and comprehensive evaluations of PDENs-based theranostic applications remain scarce. This study fills this gap by using bibliometric techniques to systematically analyze 15 years of scientific publications. The analysis is based on data retrieved from the Web of Science Core Collection, covering studies published from 2009 to 2024. Advanced bibliometric tools and visualization techniques were utilized to ensure a rigorous and detailed analysis. The results highlight China’s dominant position, contributing 35.09% of all publications, thereby significantly influencing the research trajectory in this field. Key contributions have been made by institutions such as the Nanjing University of Chinese Medicine, the Chinese Academy of Sciences and Zhejiang University, with Stefania Raimondo emerging as the most productive researcher. *International Journal of Molecular Sciences* stands out as the journal with the largest number of publications in this area. The study also identifies key related diseases, including colonic diseases, vascular diseases, osteosarcoma and DNA virus infections, etc. In conclusion, this study offers a detailed assessment of advancements and evolving patterns in PDENs-based theranostics over the past 15 years. It emphasizes critical areas that require further focus and systematic exploration by the scientific community. Additionally, this analysis identifies major research hotspots and emerging boundaries, providing scholars and research institutions with strategic insights to shape future studies.

## Graphical abstract

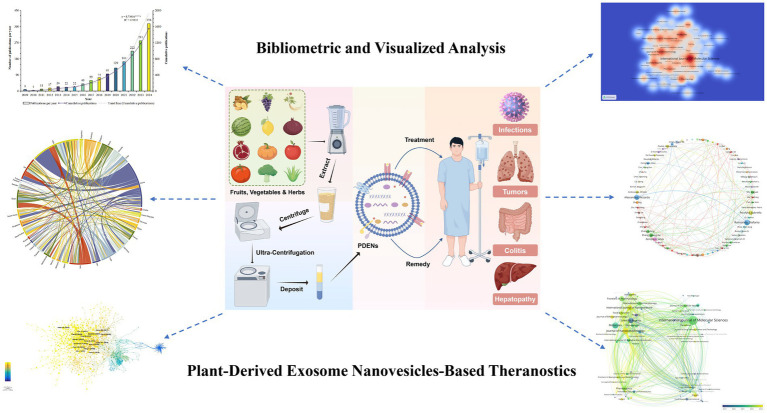

## 1 Introduction

**Figure 1:**
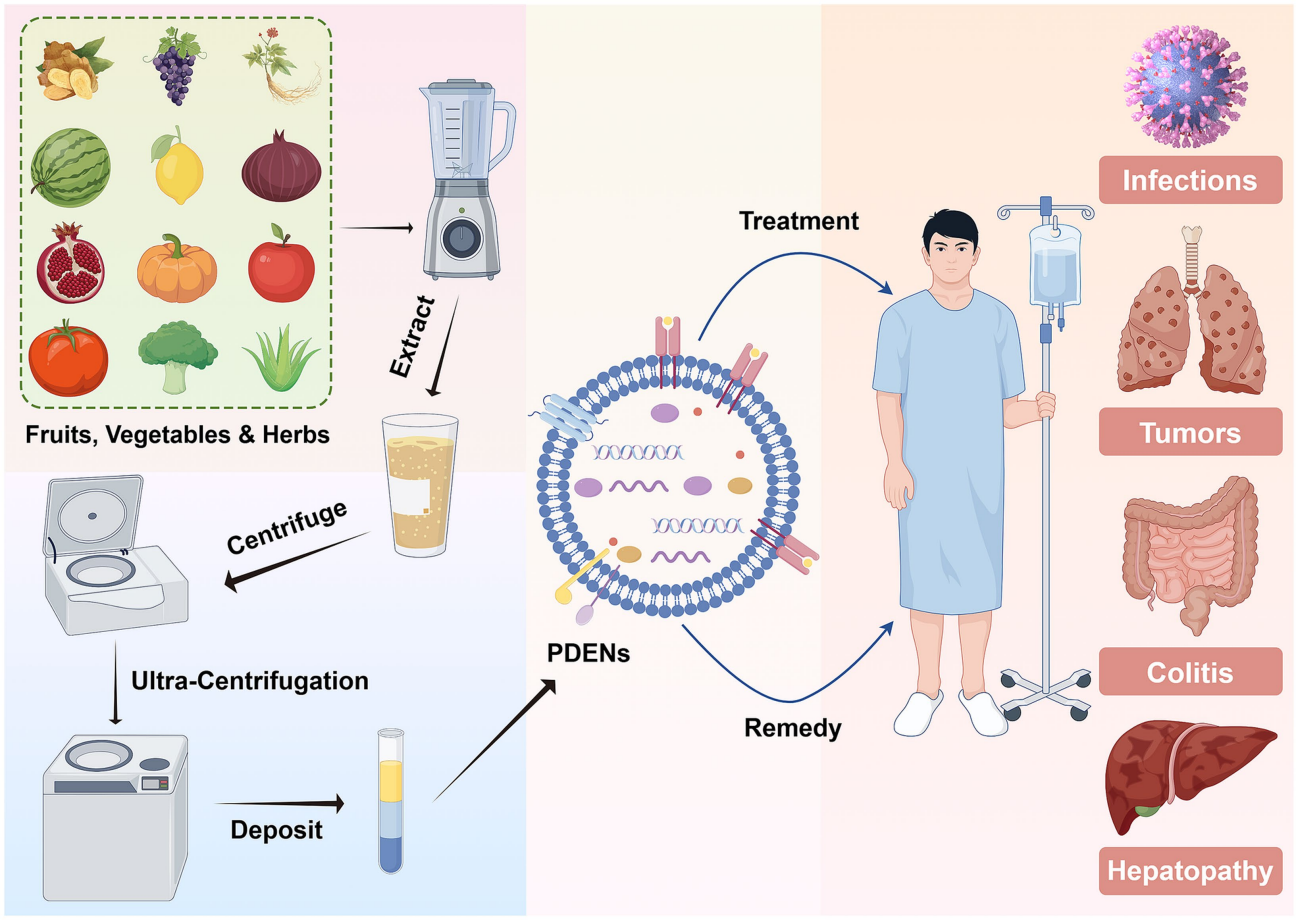
Isolation, characterization, and bioactive applications of plant-derived exosome nanovesicles. This figure was created using Figdraw (https://www.figdraw.com/static/index.html#/). PDENs, plant-derived exosome nanovesicles.

Plant-derived exosome nanovesicles (PDENs) are nanoscale particles, typically under 200 nm in diameter, secreted by plant cells (1). PDENs were first identified in the 1960s, when Halperin and colleagues observed extracellular vesicles resembling multivesicular bodies in carrot cell suspension cultures (2). Employing electron microscopy, researchers conducted a detailed morphological and structural characterization of these vesicles, thereby establishing a foundational framework for future PDEN investigations. In 2009, Mariana Regente and her colleagues successfully isolated exosome-like vesicles, ranging from 20 to 200 nm in diameter, from sunflower plants. Through the application of transmission electron microscopy and proteomic profiling, they detected these vesicles in sunflower seedlings and hypothesized their involvement in intercellular signaling processes (3). Initial investigations established fundamental methodologies for isolating and characterizing PDENs, laying the groundwork for subsequent research. Over recent years, the convergence of nanotechnology, biochemistry, and molecular biology has led to substantial refinements in PDEN separation, purification, and identification techniques. These methodological advancements have facilitated more precise analyses, thereby enhancing comprehension of their biological roles and expanding their prospective biomedical applications. With growing recognition of their therapeutic potential, PDENs have exhibited promising efficacy in addressing various pathological conditions, including colitis, hepatic disorders, malignancies, and bacterial infections (Figure 1) (4–9). This therapeutic efficacy combines the versatility of nanotechnology with the healing properties of botanical ingredients (10, 11). In addition to their therapeutic uses, PDENs have been recognized as biologically derived drug delivery systems with significant advantages over conventional methods (12). Compared to synthetic or mammalian-derived nanovesicles, PDENs exhibit enhanced biocompatibility, stability, biodistribution, circulation time, and cellular uptake (13, 14). Nanovesicles derived from mammalian sources encapsulate a diverse array of species-specific proteins, lipids, and nucleic acids, facilitating precise bioactive modulation and targeted cargo delivery (15, 16). These distinctive attributes have positioned them at the forefront of investigations in oncology, immunomodulation, and regenerative therapies. Nevertheless, their transition to clinical applications remains hindered by several critical barriers, including substantial production costs, potential immunogenic responses, and the inherent risk of pathogen transmission (17–19). In contrast, plants, including herbs, fruits, vegetables, and other botanicals, provide an eco-friendly and sustainable source, with lower immunogenicity and minimal biosafety concerns for PDENs (20, 21). They enable large-scale production with minimal environmental impact, earning the term “nano-factories” for future therapeutic systems (22, 23). Furthermore, PDENs are enriched with distinct bioactive constituents, including plant-derived microRNAs, flavonoids, and polysaccharides. These molecular components play a crucial role in mediating their anti-inflammatory, antioxidant, and anti-tumor properties (24, 25). These unique characteristics underscore the immense potential of phytonanotechnology in biomedical applications (26–29).

Recent years have seen remarkable progress in the field of PDENs, with many reviews on their biomedical applications appearing in leading academic journals (1, 13, 27, 30–41). Nonetheless, such reviews frequently exhibit inherent constraints that may undermine their impartiality. First, disciplinary biases often shape the selection of literature, leading authors to prioritize research within their own domain while disregarding valuable interdisciplinary insights. For instance, pharmacological assessments predominantly emphasize therapeutic outcomes, whereas materials science discussions center on nanoparticle physicochemical properties. Second, the reliance on narrative synthesis inherently entails selective literature inclusion, which tends to amplify the prominence of widely cited works while potentially neglecting nascent or underrepresented research areas. Consequently, these variations in analytical scope hinder a comprehensive delineation of the research landscape. The absence of a systematic framework further complicates the identification of pivotal research themes and emergent trajectories, ultimately impeding the recognition of fundamental knowledge gaps. Earlier bibliometric investigations concerning the application of PDENs in malignant tumor therapy (42) have largely concentrated on isolated therapeutic scenarios rather than offering a comprehensive perspective on their role across the broader healthcare spectrum. This constrained scope impedes a thorough grasp of evolving research trends, pivotal contributors, and emerging biomedical applications. Moreover, existing studies fail to systematically evaluate the interdisciplinary dimensions of PDENs-related research. The lack of a well-defined bibliometric framework capable of delineating publication trajectories, thematic research clusters, and collaborative scientific networks restricts the ability to pinpoint critical knowledge gaps and chart prospective directions for advancing PDENs-based theranostic strategies. To address these gaps, this study employs bibliometric analysis to investigate the development of PDENs-based theranostic applications in healthcare over the past 15 years, incorporating detailed visual data. Unlike traditional narrative reviews and meta-analyses, which synthesize findings from a predetermined corpus of studies, bibliometric and visualization-based analyses provide a more data-driven and objective means of evaluating the broader research landscape. Through the application of advanced methodologies, including co-citation network analysis, keyword co-occurrence mapping, and citation burst identification, this study offers a structured and systematic exploration of evolving research trajectories, influential thematic clusters, and the intricate web of interdisciplinary linkages shaping PDENs-based theranostics. Advanced visualization tools such as CiteSpace and VOSviewer facilitate the conversion of raw bibliometric data into structured representations, unveiling thematic clusters, keyword relationships, and citation linkages. By employing these tools, researchers can discern latent trends, interdisciplinary intersections, and the underlying structure of scientific collaborations, including institutional affiliations and thematic evolution—dimensions that traditional narrative reviews and meta-analyses may fail to capture. Moreover, bibliometric methodologies provide standardized indicators, such as citation burst detection and co-citation analysis, enabling a rigorous assessment of the impact exerted by specific publications, authors, and research institutions. This data-centric approach ensures a more equitable evaluation of scholarly contributions across various domains. By quantifying research influence and mitigating the subjective biases inherent in conventional reviews, bibliometric analysis offers a comprehensive perspective on the PDENs research landscape. These analytical insights empower scholars and policymakers to make informed decisions regarding funding allocations, recognize emergent research directions, and cultivate interdisciplinary collaborations. Through the synergy of bibliometric and visualization-based methodologies, this study not only delineates the current state of PDENs research but also highlights critical knowledge gaps and future research trajectories that qualitative assessments might overlook. Such findings underscore the necessity of systematic, large-scale evaluations in a rapidly evolving field, reinforcing a data-driven framework for advancing PDENs-based theranostics.

This study addresses four fundamental research questions: (1) What are the prevailing trends in utilizing PDENs for theranostic applications? (2) Which nations, academic institutions, and scholars are at the forefront of advancements in this particular research area? (3) Which publications, references, and keywords are most influential? (4) What are the primary diseases examined in these studies? To answer these questions, this study adopts a holistic methodology, integrating quantitative and qualitative analyses. The quantitative analysis examines research topics, publication timelines, language usage, journal representation, and the geographical and institutional demographics of authors and citations. The qualitative analysis employs keyword co-occurrence techniques to map thematic patterns in the field. This study aims to construct a comprehensive knowledge framework to assist interdisciplinary researchers and guide newcomers in identifying emerging opportunities in this rapidly evolving field. To our knowledge, this is the first bibliometric study to comprehensively examine PDENs applications across the entire healthcare sector.

## 2 Materials and methods

### 2.1 Data source and retrieval strategy

The Web of Science Core Collection (WoSCC) was selected as the primary data source for this bibliometric analysis because of its distinctive strengths. First, the interdisciplinary nature of PDENs’ theranostic applications, spanning clinical medicine, biology, and materials chemistry, requires a database that integrates diverse subject areas for a comprehensive analysis. Second, the WoSCC’s inclusion of cited references supports the creation of detailed knowledge maps (43), enabling deeper analysis of interconnected PDENs research. Third, its citation reports provide reliable benchmarks, enhancing the validity of bibliometric results. Additionally, the WoSCC offers advanced bibliometric tools that enhance document classification accuracy while maintaining superior data quality and consistency compared to other databases (44, 45). The Science Citation Index Expanded enables precise tracking of scientific progress and detailed publication trend analysis (44, 46–48), adhering to high-quality standards. Finally, the WoSCC’s journal selection methodology, guided by Bradford’s and Garfield’s Laws (43), ensures the inclusion of essential publications while minimizing bibliometric gaps. These features establish the WoSCC as an ideal resource for bibliometric research, providing reliable insights into publication patterns and emerging academic priorities. Therefore, the WoSCC has been regarded by many researchers in the industry as the most suitable database for bibliometric analysis (49–51).

This investigation performed an extensive search within the WoSCC database to gather primary research articles and review papers concerning the theranostic applications of PDENs in healthcare. The analysis covered publications from January 1, 2009, to December 2, 2024, corresponding to the seminal 2009 study by Mariana Regente et al., which identified exosomes from sunflower seeds using transmission electron microscopy and proteomic methods (3). To ensure precise and relevant results, the search strategy used a combination of Medical Subject Headings (MeSH) terms and carefully selected keywords. Keywords were classified into three main categories: (1) plant-related terms (e.g., “plant,” “herbal,” “botanical,” “Traditional Chinese Medicine”), (2) extracellular vesicle-related terms (e.g., “exosome,” “extracellular vesicle,” “nanovesicle,” “microvesicles”), and (3) theranostic and biomedical application terms (e.g., “pharmac*,” “biomedic*,” “drug deliver*,” “therapeutics,” “cancer”). These categories were systematically combined through Boolean logic (#1 AND #2 AND #3) to refine search parameters, thereby ensuring a targeted emphasis on PDENs within theranostic and biomedical research. To further optimize specificity, the search methodology underwent an initial validation process, involving a manual examination of a preliminary dataset. This evaluation facilitated the identification of potential oversights and overgeneralizations, prompting iterative adjustments to enhance accuracy. The methodology underwent multiple iterations, refined through the collective expertise of three researchers to ensure accuracy and reliability. Details of the search strategy are provided in the Supplementary materials.

### 2.2 Inclusion and exclusion criteria

The inclusion criteria targeted studies on the theranostic applications of PDENs in healthcare, focusing on original research articles and review papers in English. Exclusion criteria eliminated dissertations, letters, commentaries, editorials, conference abstracts, and articles from journals unrelated to the research topic. The research team held discussions to finalize the inclusion and exclusion criteria, ensuring consistency and relevance.

### 2.3 Data analysis

The dataset utilized in this research was sourced from the WoSCC database and systematically organized with Microsoft Excel (Office 365, Microsoft). A suite of specialized tools was employed for data analysis, including VOSviewer 1.6.18 (Leiden University, Netherlands), CiteSpace 6.3. R1 (Chaomei Chen, China), Pajek 5.16 (University of Ljubljana, Slovenia), Scimago Graphica 1.0.35 (United States),^1^ and the chorddiag R package (R Studio, version 4.2.0).

To illustrate collaboration networks across countries and regions, chord diagrams were constructed using VOSviewer and the chorddiag R package. Co-occurrence analyses of countries/regions, institutions, authors, journals, research areas, keywords, and diseases were conducted with the aid of VOSviewer, Pajek, and Scimago Graphica. CiteSpace played a pivotal role in generating detailed visualizations, such as co-citation networks, keyword distribution patterns, and relational graphs among countries/regions, institutions, authors, and journals. In addition, burst detection techniques were employed to uncover the leading 10 citation bursts for countries/regions, institutions, authors, and keywords, along with the top 20 citation bursts for co-cited references.

Insights related to diseases were derived using the Citexs Data Analysis Platform^2^, which produced visual charts that facilitated a deeper exploration of major research themes, prominent topics, and potential emerging trends in the field.

## 3 Results and discussion

### 3.1 Scholarly impact and growth trends

**Figure 2:**
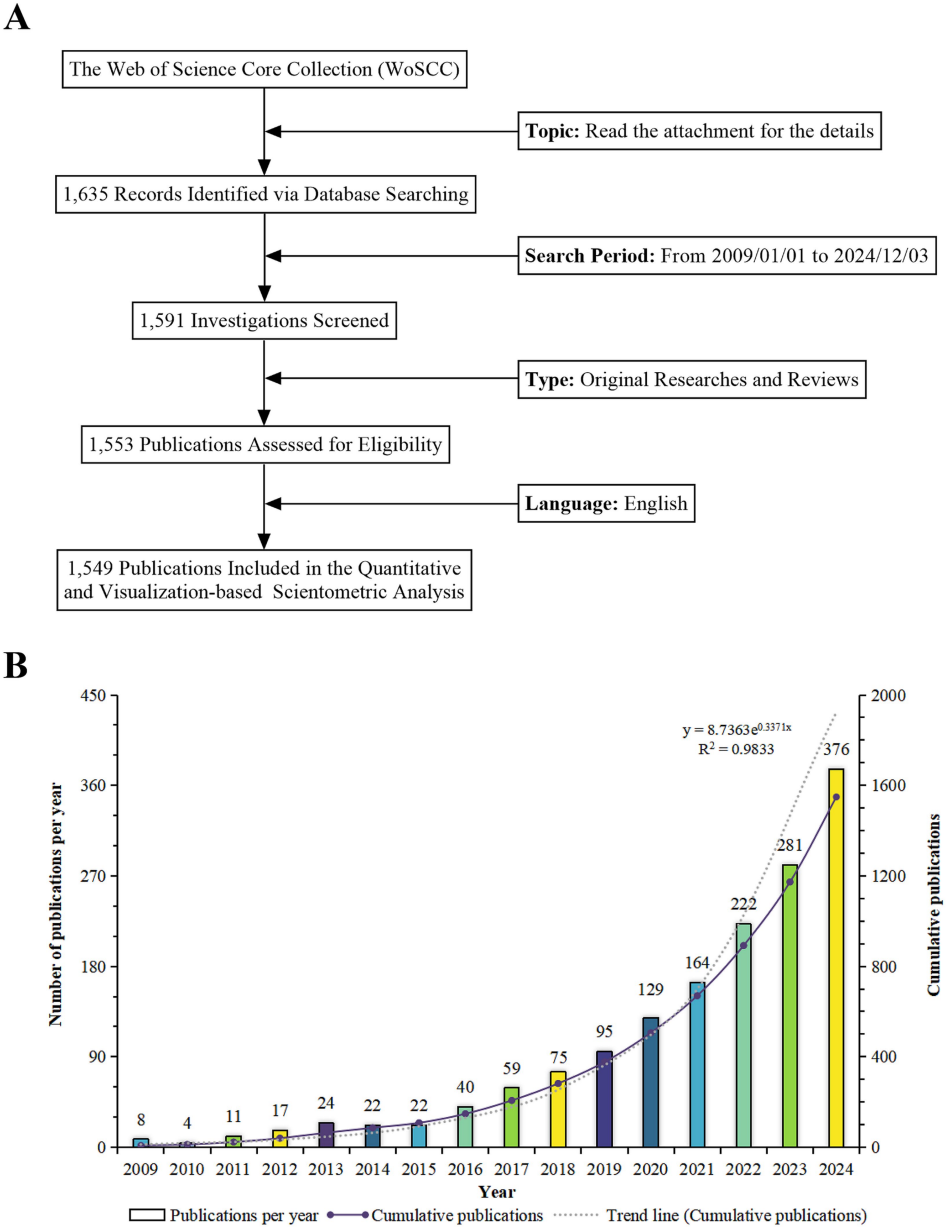
**(A)** A schematic illustration presenting the methodology employed for literature search and selection. **(B)** An analysis of the temporal tendencies in research concerning the theranostics applications of plant-derived exosome nanovesicles in healthcare from 2009 to 2024.

Figure 2A illustrates the procedure for data retrieval and compilation. The advancement of research is frequently reflected in the volume of scientific publications (52). From 2009 to 2024, a total of 1,549 publications related to the theranostic applications of PDENs in healthcare were gathered. This collection encompassed 1,101 original research articles and 448 review papers, generating an average of 103.27 publications per annum, reflecting the long-term accumulation and continuous development of research in this field. In addition to chronicling prior research endeavors, the upward trajectory of publications provides a critical foundation for guiding future investigations. The consistent growth in scholarly output reflects an expanding research landscape, encompassing both mechanistic explorations of PDENs’ therapeutic functionalities and advancements in scalable production methodologies. As scientific inquiry continues to prioritize enhancements in PDENs’ bioavailability, targeting precision, and clinical applicability, forthcoming studies can leverage these developments to tackle persisting challenges, including the standardization of isolation protocols and the refinement of bioengineering approaches. The annual number of published articles gradually rose from 8 in 2009 to 376 in 2024, signifying the increase in attention and activity in the research field of the theranostic applications of PDENs in healthcare. The period with the highest annual growth rate of the number of published articles was in 2011, attaining 175%, and the number of published articles was more than twice that of the previous year; followed by 2016, with an annual growth rate of 81.82%. To model the cumulative growth in publication volume, a polynomial function y = 8.7363e^0.3371x^ (R^2^ = 0.9833) was fitted, where x corresponds to the number of years and y indicates the total number of publications (Figure 2B). The excellent goodness of fit implies that this function offers an accurate portrayal of the increasing publication output in the field. These findings spotlight the growing interest in the theranostic applications of PDENs in healthcare and predict continued substantial growth in the forthcoming years. A key advantage of the cumulative growth model lies in its ability to forecast emerging research frontiers. Through trend extrapolation, this model serves as a strategic tool, guiding researchers in directing their efforts toward prospective areas of significant scientific influence.

The rapid expansion of PDENs-related research is shaped by several key determinants, including financial investment, regulatory evolution, and industrial participation. First, funding plays a foundational role in driving progress. Both governmental agencies and private-sector stakeholders are progressively allocating substantial resources to PDENs research, recognizing its disruptive potential in biomedical innovation. For instance, initiatives spearheaded by the National Natural Science Foundation of China (NSFC) and equivalent international organizations have significantly advanced developments in this field. Competitive grants and innovation-focused funding programs have facilitated cross-disciplinary collaborations, accelerating the transition from fundamental research to clinical application. Second, regulatory policies serve as a crucial mediator in the clinical translation of PDENs. Regulatory bodies are continually refining evaluation frameworks to assess the safety, efficacy, and large-scale production feasibility of PDENs-based technologies. The implementation of standardized procedures and well-defined approval mechanisms will streamline commercialization, ensuring a more seamless transition from experimental research to therapeutic deployment. Third, the commercial sector is increasingly engaged in PDENs research. Recognizing their potential in drug delivery and theranostic applications, pharmaceutical and biotechnology enterprises are amplifying investments in research and development. Collaborative ventures between industry and academia further accelerate the transformation of PDENs-based innovations into viable biomedical solutions. Collectively, these interrelated factors drive the sustained momentum of PDENs research, reinforcing its growing prominence in the healthcare sector.

The sharp increase in research activity, as demonstrated by the annual publication growth, indicates the necessity for targeted funding initiatives and strategic policy frameworks to support innovation in PDENs-based theranostics. Policies should concentrate on fostering interdisciplinary collaborations and stimulating translational research to accelerate clinical adoption. The predictive growth model suggests long-term significance, encouraging continuous investment and the establishment of regulatory pathways for PDENs-based products. The increase in publication volume reveals the field’s dynamic nature and the escalating global attention it receives. The cumulative growth model (*R*^2^ = 0.9833) provides a reliable forecast of future research trends, guiding researchers to prioritize influential and innovative directions. Furthermore, the considerable growth rates in specific years mirror breakthrough moments, motivating researchers to investigate underlying factors and explore new frontiers in PDENs-based theranostics. These emerging patterns underscore the necessity of refining research methodologies to align with the fast-paced evolution of scientific inquiry. As advancements in adjacent disciplines, including nanomedicine and extracellular vesicle engineering, continue to influence the trajectory of PDENs research, experimental frameworks must maintain a high degree of adaptability. The integration of cutting-edge technologies and interdisciplinary perspectives will be essential for fostering sustained innovation in the field. In addition to offering insights into historical and contemporary research trends, the predictive model functions as a strategic guide, directing future experimental investigations and optimizing resource distribution. The vigorous growth of publications implies an expanding knowledge base that industry practitioners can utilize to develop state-of-the-art PDENs-based therapeutic and diagnostic products. The predictive model offers insights into future research activity, assisting companies in aligning their research and development strategies with emerging trends. Additionally, the long-term accumulation of knowledge highlights the feasibility and growing commercial potential of PDENs-based technologies, paving the way for investments in scalable manufacturing, clinical trials, and market introduction.

### 3.2 Performance analysis

#### 3.2.1 Global collaboration and productivity

**Figure 3:**
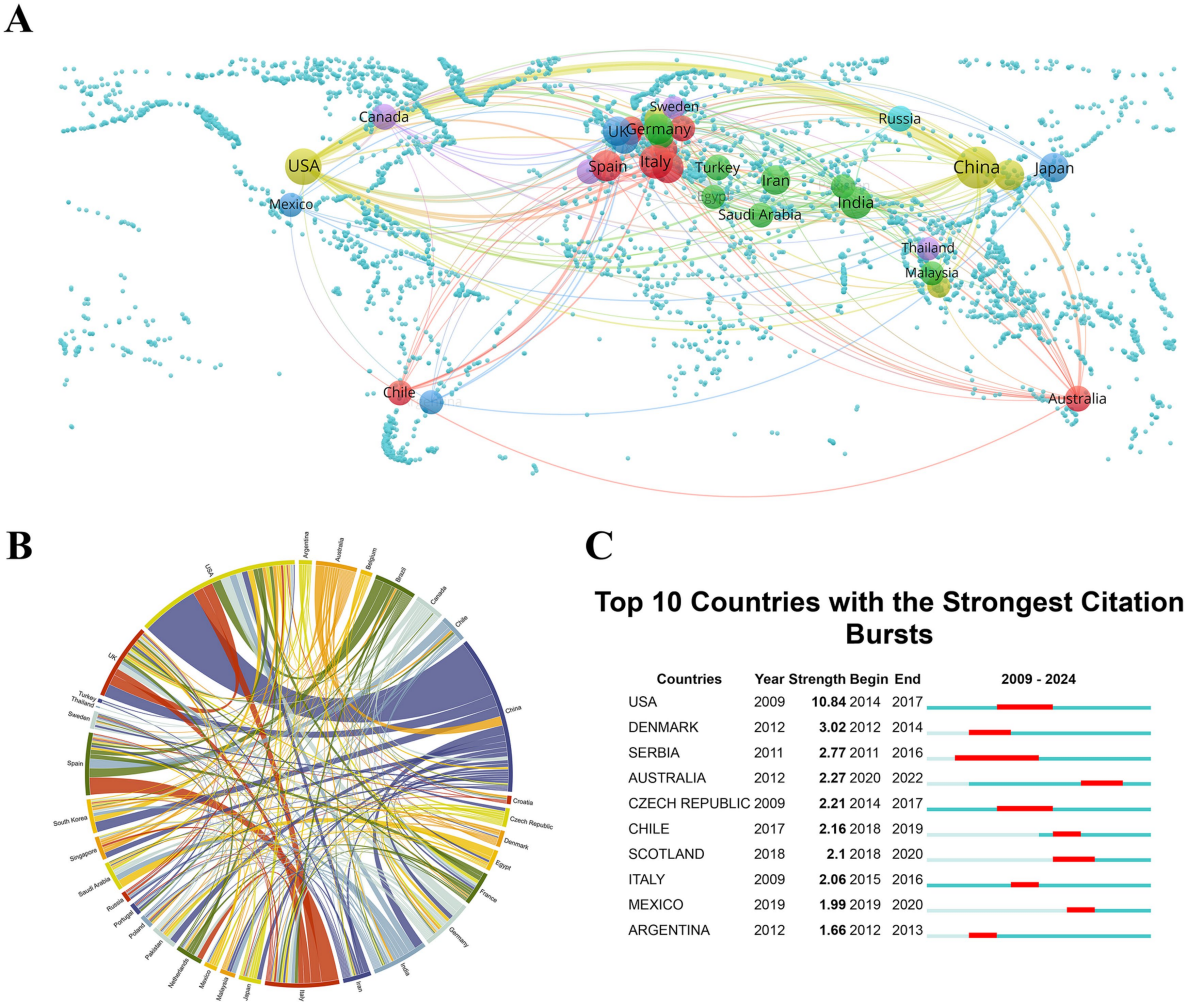
**(A)** A global display of the theranostics applications of the plant-derived exosome nanovesicles (PDENs) in the healthcare domain, with spheres representing different countries. The size of each sphere corresponds to the volume of publications, while the thickness of the connecting lines shows the intensity of collaborations among nations. **(B)** Chord diagrams portraying international partnerships in the application of the PDENs within biomedical research, where the thickness of the lines indicates the strength of collaborative efforts between countries. **(C)** Research outputs from the 10 leading countries in the theranostics applications of PDENs for biomedical research, highlighted in red to indicate a significant increase in publication rates. The term “Burst” refers to a sudden escalation in research activity, with “BurstBegin” and “BurstEnd” denoting the onset and tapering off of this surge, respectively.

Research on the theranostic applications of PDENs in healthcare encompasses 84 countries/regions. Figures 3A,B portray national collaboration networks, highlighting the contributions of countries having a minimum of 10 publications. These findings offer valuable viewpoints on potential routes for strategic collaborations and the facilitation of knowledge exchange (53). Among the total 1,549 publications, China contributes 35.09%, a figure nearly seven times higher than that of Spain, which ranks fifth in publication volume. China’s dominant share of research output underlines a national policy that prioritizes the advancement of healthcare technologies. The substantial investments made by the Chinese government in research and development, with a strong emphasis on innovation, play a vital role in this achievement. This support has not only enhanced domestic research capabilities but also positioned China at the forefront of global health innovation. For industry practitioners, this presents an opportunity to establish partnerships with leading academic institutions and research centers in these countries to expedite product development and market introduction. However, the dominance of a few specific countries might potentially lead to a narrowing of research topics and methods, constraining the diversity of approaches. Expanding the participation of underrepresented nations could enrich the diversity and robustness of research outcomes and contribute to a more inclusive scientific environment, fostering novel approaches and methodologies in the field. After China, the United States, Italy, and India are the subsequent leading countries in terms of publication volume, with 225, 149, and 94 papers, respectively. Among the top 25 countries by publication volume, Pakistan distinguishes itself with the highest average citations per paper at 63.47, closely trailed by the United States at 56.03. These findings emphasize the significance of fostering global collaboration, diversifying research participation, and balancing quantity with quality to promote the theranostic applications of PDENs in healthcare and drive influential innovation. In addition to enhancing scholarly output, global research collaborations play a crucial role in expediting the clinical application of PDENs-related advancements. Such partnerships contribute to the unification of experimental methodologies, support the implementation of multinational clinical trials, and drive regulatory alignment—essential processes for converting fundamental laboratory findings into practical medical interventions. By integrating expertise from diverse geographical regions, these cooperative efforts facilitate the seamless transition from basic scientific exploration to clinical deployment, thereby improving the accessibility of PDENs-based therapeutic innovations for patients worldwide.

In the chord diagram, the outer arcs correspond to various countries and regions, with the length of each arc reflecting the publication volume associated with that region (54). The extent of national collaboration is reflected in the connections among these arcs. The extensive international cooperation indicates a global endeavor to advance healthcare through nanotechnology, offering valuable insights for policymaking related to research funding and international collaboration. Among the collaborative relationships, the strongest connection is witnessed between China and the United States, with a link strength of 50, which underscores the value of fostering bilateral agreements to accelerate innovation. Policymakers can utilize these insights to allocate funding for joint projects, incentivize multinational research initiatives, and promote global knowledge exchange. Subsequently, the link strength between Italy and Spain amounts to 15. In the third place is the link strength between China and the United Kingdom at 11 (Figure 3B). For researchers, comprehending these collaboration networks can guide decisions concerning strategic partnerships with academic institutions, thereby enhancing innovation capabilities (55). Industry professionals can utilize this information to establish research and development partnerships, gain access to state-of-the-art technologies, and foster collaborations with prominent institutions. These collaborations can accelerate the translation of research findings into marketable products. By fostering these collaborations, multidisciplinary research teams can facilitate the exchange of both preclinical and clinical datasets, optimize large-scale production processes, and tailor PDENs-based therapeutic strategies to align with varying regulatory frameworks and healthcare infrastructures. Conducting evaluations of PDENs-driven theranostic platforms across diverse medical environments allows for a more rigorous assessment of their safety and therapeutic efficacy, thereby expediting their integration into clinical practice. Additionally, identifying less robust connections may reveal opportunities to diversify and expand industrial collaborations with emerging markets and underrepresented regions. The dense networks of cooperation emphasize strategic alliances that are ready to facilitate knowledge exchange and drive innovation, accelerating the progress in this field. Recognizing the key players and their collaborative dynamics can assist in forming productive partnerships, selecting relevant conferences, and identifying potential collaborators, relevant conferences, and productive academic environments to further their careers and research impact. These findings stress the significance of fostering international collaboration for policymakers, researchers, and industry practitioners, driving innovation and accelerating the development of PDENs-based healthcare solutions.

The analysis of citation bursts offers actionable insights for prioritizing research funding and policy-making (56). These trends also highlight potential market opportunities and the development of cutting-edge technologies, allowing companies to tailor their research and development efforts to align with prominent healthcare advancements. As illustrated in Figure 3C, citation bursts for the top countries and regions are displayed, with each surge’s intensity indicated by a red line. Specifically, the United States experienced a significant rise in citations between 2014 and 2017, with the peak burst intensity reaching a value of 10.84. Serbia, Denmark, and Argentina displayed citation bursts earlier. Serbia exhibited the longest citation burst duration, spanning from 2011 to 2016, lasting a total of 6 years. Among the top 10 countries with citation bursts, Australia experienced its citation surge at a later stage, indicating that recent research from this country has gained considerable attention in the field of theranostic applications of PDENs in healthcare. Companies can strategically make investments in research and development partnerships, licensing agreements, or recruitment efforts in these areas to align with emerging healthcare trends and maintain a competitive edge. By understanding these trends, researchers can better position themselves to take advantage of and contribute to the rapid developments in this transformative field, obtain funding, and publish in high-visibility areas, thereby enhancing their academic and professional status.

#### 3.2.2 Core institutions and collaborative networks

Analyzing the patterns of institutional collaborations provides crucial insights for industry professionals, helping them identify potential research partners and synchronize their product development efforts with leading-edge scientific advancements. Collaborating with these institutions offers researchers improved access to funding, state-of-the-art facilities, and interdisciplinary networks, which, in turn, fosters their involvement in pioneering innovations. Over the past 15 years, 2,181 institutions have made substantial contributions to research on the theranostic applications of PDENs in healthcare. Among these, Nanjing University of Chinese Medicine is recognized as a frontrunner in the field, with 26 publications (1.68%). This output reflects the institution’s strategic emphasis on advancing research in phytonanotechnology and healthcare, bolstered by significant government funding and support. Following closely are the Chinese Academy of Sciences (25 publications, 1.61%) and Zhejiang University (23 publications, 1.48%). National initiatives, such as the NSFC and national strategies like “Made in China 2025,” have fostered a favorable environment for the progress of nanotechnology and healthcare. However, the concentration of research output in Chinese institutions gives rise to concerns about the potential limitations on research diversity. While this concentration may drive rapid progress, it also bears the risk of narrowing the range of perspectives and methods, which could impede the development of alternative approaches and innovative solutions. Policymakers can utilize this evidence to strengthen funding mechanisms, promote institutional partnerships, and encourage a more diverse range of research perspectives by supporting underrepresented regions and institutions. This approach ensures sustainable and balanced growth in nanotechnology and healthcare research.

**Figure 4:**
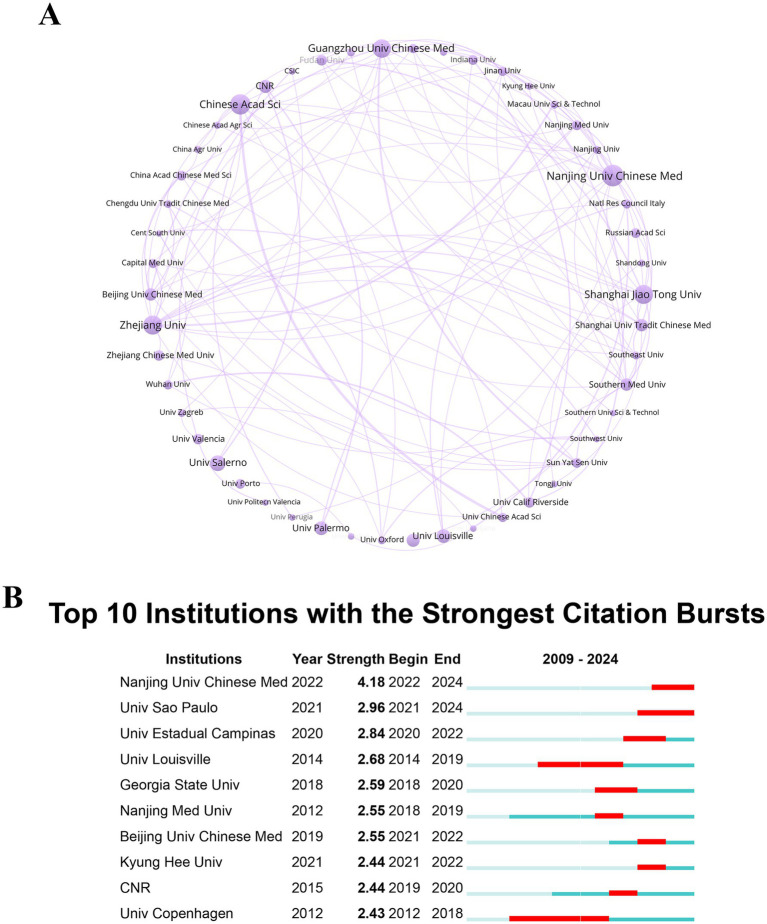
**(A)** Institutional co-occurrence analysis map. The combination of spheres and text constitutes an entity, denoting an institution; the connecting lines between circles represent collaborative occurrences among institutions; the thickness of the lines shows the strength of collaboration between institutions; the size of the circles is positively correlated with the number of publications from the institutions. **(B)** Citation bursts related to the leading 10 institutions, highlighted with red bars to indicate periods of increased citation frequency. The term “Burst” indicates a rapid elevation in the visibility of a topic, while “BurstBegin” and “BurstEnd” denote the commencement and tapering off of this increased activity, respectively.

A network map was developed to illustrate the collaborative interactions among research institutions, with a threshold of 8 publications per institution established to filter the data (Figure 4A). This map acts as a valuable tool for researchers, providing insights into potential collaborations and encouraging interdisciplinary and inter-institutional partnerships that can elevate the quality of research. Each institution is depicted as a combination of a sphere and text, with lines between them denoting collaborative relationships. The thickness of these lines is proportional to the strength of the collaboration, while the size of each circle corresponds to the institution’s publication volume. Zhejiang University stands out with the highest total link strength (23), indicating its considerable engagement in collaborative efforts. Among the various institutional pairs, the highest collaboration intensity is observed between the Chinese Academy of Sciences and the University of Chinese Academy of Sciences, suggesting an exceptionally strong and frequent partnership. Following this, other notable collaborations include those between Guangzhou University of Chinese Medicine and Sun Yat-sen University, as well as between Zhejiang University and Macau University of Science and Technology. Understanding these collaborative dynamics enables researchers to identify potential partners and prioritize interdisciplinary collaborations. Institutional collaborations play a crucial role in driving advancements in PDENs-based theranostics by fostering resource integration, interdisciplinary synergy, and technological dissemination. Prominent institutions, including Zhejiang University and the Chinese Academy of Sciences, serve as central nodes for knowledge exchange, expediting the transition from foundational research to clinical implementation. These cooperative efforts enable researchers to synthesize expertise spanning nanomaterial engineering, drug delivery mechanisms, and biomedical sciences, thereby expanding the research landscape of PDENs. Furthermore, alliances between academic institutions and medical centers strengthen the clinical applicability of PDENs-driven diagnostics and therapeutics, streamlining their adoption within healthcare systems. Establishing strategic inter-institutional partnerships can further refine experimental methodologies, optimize funding distribution, and shape policy directives to sustain long-term innovation. Such partnerships can enhance access to funding, resources, and expertise, ultimately boosting the quality and scope of their research. Furthermore, the map can guide researchers in targeting conferences and academic networks where these collaborations are most active. Policymakers can utilize this collaboration data to foster strategic alliances between established and emerging institutions, thereby enhancing the research ecosystem as a whole. By incentivizing collaborative projects through targeted funding and policies, they can bridge gaps in research capacities and create a more balanced and innovative research ecosystem. Additionally, encouraging cross-regional collaborations could further enhance the global impact of PDENs-based healthcare research.

Identifying institutions experiencing citation bursts offers valuable insights for shaping policies that foster high-impact research and promote innovation in healthcare (57). Additionally, recognizing such institutions assists businesses in spotting emerging market trends and technologies, enabling more targeted investment and recruitment strategies in areas with high growth potential. Through the use of CiteSpace analysis (Figure 4B), this study has pinpointed institutions that have experienced significant increases in citation counts. Nanjing University of Chinese Medicine witnessed a sharp increase from 2022 to 2024, with the highest burst intensity of 4.18. Among the 10 leading institutions, the University of Copenhagen experienced the earliest citation burst, with the longest duration, spanning 7 years. This suggests that the research from this institution not only began earlier but also maintained a significant academic impact on the theranostic applications of PDENs in healthcare over an extended period. In contrast, Nanjing University of Chinese Medicine and Universidade de São Paulo are the institutions that have seen citation bursts in the past 2 years, indicating that their recent research has garnered considerable attention in the field of PDENs’ theranostic applications in healthcare. Policymakers can utilize this information to allocate resources and funding strategically, supporting emerging research hotspots with high growth potential. By collaborating with or benchmarking against these institutions, researchers can align their work with cutting-edge trends in PDENs theranostic applications. The analysis also reveals shifts in academic attention, helping researchers focus on rapidly developing areas and enhance their visibility by engaging in high-interest topics at leading institutions.

#### 3.2.3 Collaboration dynamics among core authors

**Figure 5:**
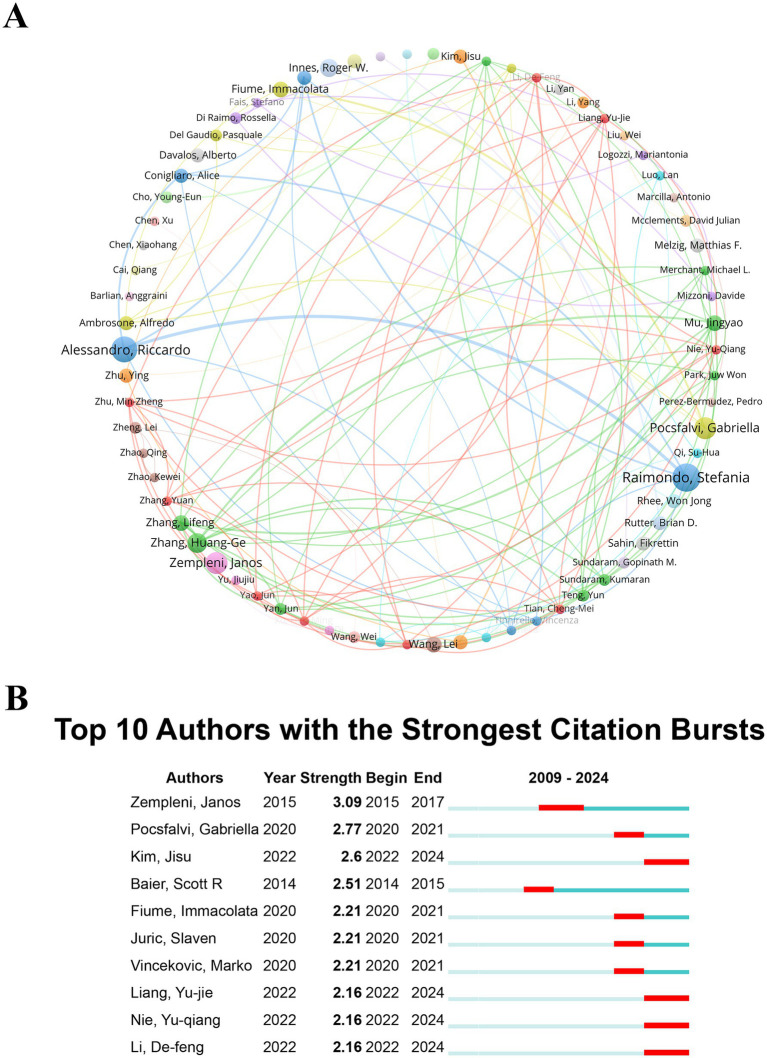
**(A)** A co-occurrence map of authors, with nodes presented as circles and accompanied by text labels, where diverse colors indicate different clusters. **(B)** The 10 authors showing the highest citation bursts in the field of “the applications of plant-derived exosome nanovesicles in healthcare.” The term “Burst” indicates a rapid increase in research attention during a specified time period, with “BurstBegin” marking the start of this surge and “BurstEnd” indicating its eventual decrease.

Recognizing leading authors and key research clusters is crucial for guiding policymakers in allocating funds to areas with significant impact, ensuring that resources are directed toward the most active and influential networks in theranostics research. The network of author collaborations plays a pivotal role in advancing PDENs-based theranostics by bringing together specialists from diverse subfields. Moreover, strong collaborative ties frequently lead to high-impact publications and the emergence of influential research clusters, which serve as foundational references for new scholars in the field. Enhancing these collaborative frameworks may provide valuable strategic insights for directing future PDENs research, promoting continuous innovation, and advancing scientific progress. Researchers who have accumulated a significant number of citations and maintain a steady publication output provide critical perspectives that can shape the trajectory of future investigations. For companies pursuing innovation in theranostics applications, being informed about top researchers and their collaborative networks is essential for identifying potential research and development partners, thereby obtaining access to cutting-edge knowledge and technology. A thorough examination of authorship within the realm of PDENs in theranostics revealed a total of 8,440 contributors, among which five researchers distinguished themselves by publishing a minimum of 10 articles each. These scholars have made considerable contributions to the research landscape, and their established networks present potential strategic partnerships. The visualization of author networks and co-authorship data enables emerging researchers to identify influential figures and create focused collaboration opportunities, accelerating research progress. To further explore collaboration patterns, VOSviewer was utilized to produce visual representations, with a minimum threshold of 5 publications per author. The visualizations produced illustrate that the size of each node is proportional to the publication count of the corresponding author, with distinct colors used to classify authors into various groups (Figure 5A). The strength of the links between nodes represents the intensity of collaborative relationships. Notably, 70 authors surpassed the publication threshold. The total link strength values of Mu Jingyao, Zhang Huang-Ge, and Zhang Lifeng (total link strength = 49) are tied for first place, indicating that these authors have the strongest willingness to collaborate with others. The thickness of the lines between the circles represents the collaboration intensity among the authors. The collaboration intensity value between Riccardo Alessandro and Stefania Raimondo is the highest (12), indicating that these two authors collaborate most frequently and have the closest collaboration relationship. Stefania Raimondo leads in publication count with 14 papers, closely followed by Riccardo Alessandro with 13. Gabriella Pocsfalvi and Janos Zempleni share the third position, each having 11 publications. This suggests that these authors have a high level of concern and produce more in the field of the theranostic applications of PDENs in healthcare.

Recognizing leading authors such as Stefania Raimondo and Riccardo Alessandro enables targeted allocation of funds to influential research groups. This ensures that resources are channeled toward areas with the greatest potential for breakthrough innovations. Furthermore, identifying collaborative clusters assists in formulating policies that encourage international and interdisciplinary partnerships. These are vital for addressing complex healthcare challenges. By prioritizing high-collaboration-intensity networks, policymakers can create environments that stimulate knowledge exchange and maximize the return on public research investments. For emerging researchers, the analysis functions as a practical guide in the competitive field of PDENs research. Identifying influential figures and high-collaboration-intensity networks empowers early-career scholars to strategically align with prominent clusters. This boosts their academic visibility and expedites their contributions. This roadmap is particularly valuable for promoting cross-disciplinary collaborations, as the field of theranostics requires the integration of expertise from materials science, medicine, and biotechnology. Additionally, comprehending the structure of these networks enables researchers to identify gaps and underexplored areas, uncovering opportunities for innovative contributions. The study provides industry practitioners with actionable insights for identifying potential research and development partners. Authors with high total link strength, such as Mu Jingyao, Zhang Huangge, and Zhang Lifeng, offer strategic opportunities for accessing cutting-edge knowledge and technology. Companies pursuing innovation in theranostics can utilize these insights to form partnerships with high-performing researchers and their networks, ensuring early access to groundbreaking developments. Furthermore, the study underlines the significance of collaboration intensity as a measure of reliability and synergy, guiding industries to invest in stable and productive academic-industry partnerships.

Analyzing citation bursts provides essential insights into the frequency and intensity of citations received by authors within a particular academic discipline over a defined time frame. Figure 5B illustrates the top 10 authors who experienced the most significant citation bursts in the area of theranostic applications of PDENs in healthcare. The data reveals that Janos Zempleni experienced a citation surge between 2015 and 2017, with the peak intensity value reaching 3.09. Scott Baier began to have a citation burst the earliest, and the burst period was between 2014 and 2015, suggesting that this author was active in the field of the theranostic applications of PDENs in healthcare earlier. The authors who had citation bursts in the past 2 years are Kim Jisu, Liang Yujie, Nie Yuqiang, and Li Defeng. The burst periods were all from 2022 to 2024, indicating that the research results of these authors have received high attention in the field of the theranostic applications of PDENs in healthcare in the last 2 years. This approach furnishes policymakers, researchers, and industry stakeholders with actionable insights to prioritize investments, collaborations, and technological advancements, ultimately driving the growth and application of PDENs in healthcare. The identification of citation bursts enables policymakers to track influential research trends and prioritize funding to support active and impactful researchers. The temporal patterns also assist researchers in understanding the evolution of the field and identifying gaps for innovative exploration. By analyzing burst trends, companies can strategically position themselves to collaborate with the most influential researchers, accelerating innovation pipelines and gaining competitive advantages.

### 3.3 Journals, related fields, and co-cited references

#### 3.3.1 Analysis of journals and related fields

**Figure 6:**
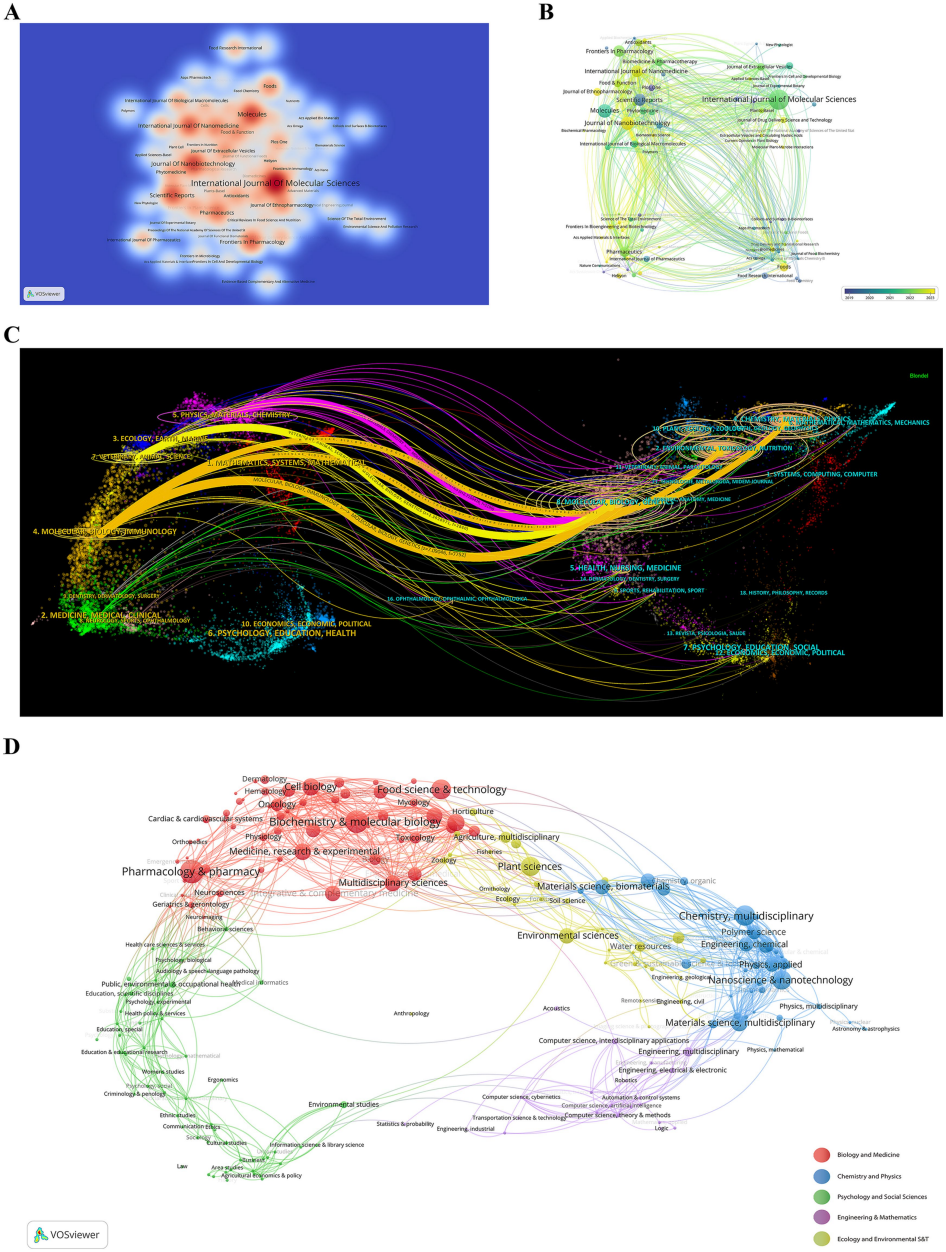
**(A)** A density visualization map depicting journal citations, where the intensity of color reflects the volume of publications. **(B)** A journal distribution map arranged by the average year of publication, with blue representing earlier years and yellow indicating more recent ones. The size of each node corresponds to the frequency of keywords, and the gradient of circle colors indicates the year of publication. **(C)** A dual-map overlay presenting citation networks among journals focusing on the theranostics applications of the plant-derived exosome nanovesicles in the healthcare domain. Each point represents a journal, while the connecting curves show citation relationships, highlighting interdisciplinary trends and the evolution of citations. **(D)** An analysis of research subject areas, where distinctively colored spheres represent different academic disciplines.

The visualization of publication data across various journals offers considerable insights into the academic communication network of 679 journals that focus on publishing research related to the theranostics applications of PDENs in healthcare. A heatmap, founded on thermodynamic principles, is employed to represent those journals having at least 5 papers in their publication distribution. The color intensity displayed on the heatmap is directly related to the number of publications contributed by each journal (Figure 6A), aiding researchers in selecting suitable journals for their work. The journal with the highest publication count is the *International Journal of Molecular Sciences*, which has published a total of 47 articles. The majority of these appeared between 2021 and 2022. Subsequently, *Molecules* and *Journal of Nanobiotechnology* have published 29 and 28 papers, respectively. Their average publication times are 2021 to 2022 and 2023 respectively, suggesting that there have been more articles in the domain of the theranostic applications of PDENs in healthcare. The visualization of publication data presents a comprehensive perspective of the academic communication scenario surrounding PDENs in theranostics. By scrutinizing publication trends and journal outputs, policymakers, researchers, and industry practitioners obtain valuable insights into the most influential platforms for disseminating research. This information can direct decision-making regarding funding, collaboration, and commercialization strategies, advancing the progress of theranostics in healthcare.

The identification of prominent journals through citation metrics offers precious guidance to policymakers, researchers, and industry practitioners. This analysis helps stakeholders understand the evolving trends in theranostics research better, optimize their publication strategies, and make well-informed decisions about funding, collaboration, and product development. By using this data, stakeholders can actively contribute to and gain from the rapid progress in the theranostic applications of PDENs in healthcare. In Figure 6B, each journal is depicted by a circle. Color coding indicates the year of establishment. The size of each circle represents the number of publications associated with that journal, while the color indicates the average year of publication. Blue hues reflect earlier publications, and yellow represents more recent ones, as shown in the color gradient in the lower right corner. Based on the figure, among the top 20 journals in terms of publication volume, *Plos One* has a relatively earlier average publication period, spanning from 2018 to 2019. In contrast, *Journal of Nanobiotechnology*, *Foods*, and *Journal of Ethnopharmacology* published works in the field of PDENs theranostics in healthcare more recently. This indicates that the research in the field of the theranostic applications of PDENs in healthcare is the research hotspot and direction of these journals in recent years.

The dual-map overlay technique employed for journal analysis offers an in-depth perspective on the shifting positions of scientific research hubs and the dispersion of journals across various academic fields (58). The labels of the map show the various research areas covered by the articles. On the right side, the journals that cite other works are presented, and on the left side, the journals being cited are shown. Citation trajectories are depicted by lines in varying colors, with the thickness of these lines reflecting citation frequency, as indicated by the *z*-score scale (44). This visualization helps identify emerging trends and changes in scientific focus, allowing researchers to strategically direct their investigations. As shown in Figure 6C, the research publications in this field are mainly concentrated in the journals of Physics, Materials, Chemistry, Molecular Biology, Immunology, and Veterinary and Animal Science. The citations are focused in the journals of Chemistry, Materials, Physics, Environmental Toxicology, Nutrition, and Molecular Biology and Genetics. Each point on the graph represents a journal. The curves between the left and right parts of the graph are citation connections. The paths of these connections provide an understanding of the interdisciplinary relationships in this field and fully illustrate the origin and development of the citations. The citation landscape reveals the inherently transdisciplinary nature of PDENs research, linking foundational biological sciences with applied technological advancements. Robust citation networks between Molecular Biology, Genetics, and Chemistry highlight a strong emphasis on molecular-scale investigations, whereas linkages with Materials Science and Physics point to ongoing efforts aimed at refining nanovesicle design and delivery mechanisms. Furthermore, citations from Environmental Toxicology and Nutrition reflect a growing research focus on the safety, biocompatibility, and dietary applications of PDENs. These interdisciplinary interactions suggest that integrating advanced chemical synthesis methodologies with biomedical applications may further enhance the theranostic capabilities of PDENs. This understanding enables policymakers to identify areas needing cross-disciplinary collaboration and customize funding strategies to promote integrated research. By recognizing citation pathways and their sources, policymakers can more effectively allocate resources to journals and domains with a proven impact, ensuring a more strategic investment in healthcare innovation. By analyzing the citation pathways, researchers can track interdisciplinary connections, such as the influence of Molecular Biology and Genetics on Chemistry and Materials Science, to better position their work within these networks. This approach helps in planning publication efforts and aligning with emerging research trends in various fields. The citation trajectories also enable industries to determine which journals or research domains are driving technological advancements, allowing them to align their research and development strategies with the latest developments.

The research literature on the theranostics applications of PDENs in healthcare, sourced from the WoSCC database, was analyzed statistically and visually using VOSviewer software. The identified articles were grouped into five main thematic areas. As shown in Figure 6D, the clusters are represented by spheres of different colors, each indicating a separate research domain. The main focus of studies in this area is on the red clustered “Biology and Medicine” field and the blue “Chemistry and Physics” field. Among them, the number of articles in the segmented fields such as “Pharmacology & Pharmacy,” “Biochemistry & Molecular Biology,” “Chemistry,” and “Multidisciplinary” occupies a relatively high proportion. The identification of key thematic areas like “Biology and Medicine” and “Chemistry and Physics” emphasizes the interdisciplinary nature of research in the theranostic applications of PDENs. Policymakers can utilize this understanding to prioritize funding for these domains, promoting integrated approaches that enhance healthcare innovation. Also, the segmentation of fields into areas such as “Pharmacology & Pharmacy” and “Biochemistry & Molecular Biology” offers a roadmap for developing specialized policies to support translational research efforts. For researchers, the clustering analysis provides a structured overview of the main research domains. This enables them to identify trending topics and gaps in the literature. By concentrating on well-represented fields such as “Pharmacology & Pharmacy” and “Chemistry,” researchers can align their investigations with established areas of interest while exploring interdisciplinary opportunities, especially at the intersection of “Biology and Medicine” and “Chemistry and Physics.” Industry practitioners can take advantage of the clustering results to align their research and development strategies with high-impact areas. For instance, the prominence of “Pharmacology & Pharmacy” and “Biochemistry & Molecular Biology” suggests significant opportunities for developing new therapeutic and diagnostic applications. Understanding the segmentation of research fields helps industries identify potential collaborators and target innovations in areas where PDENs-based theranostics has the greatest market potential.

#### 3.3.2 Co-cited references and references burst

**Figure 7:**
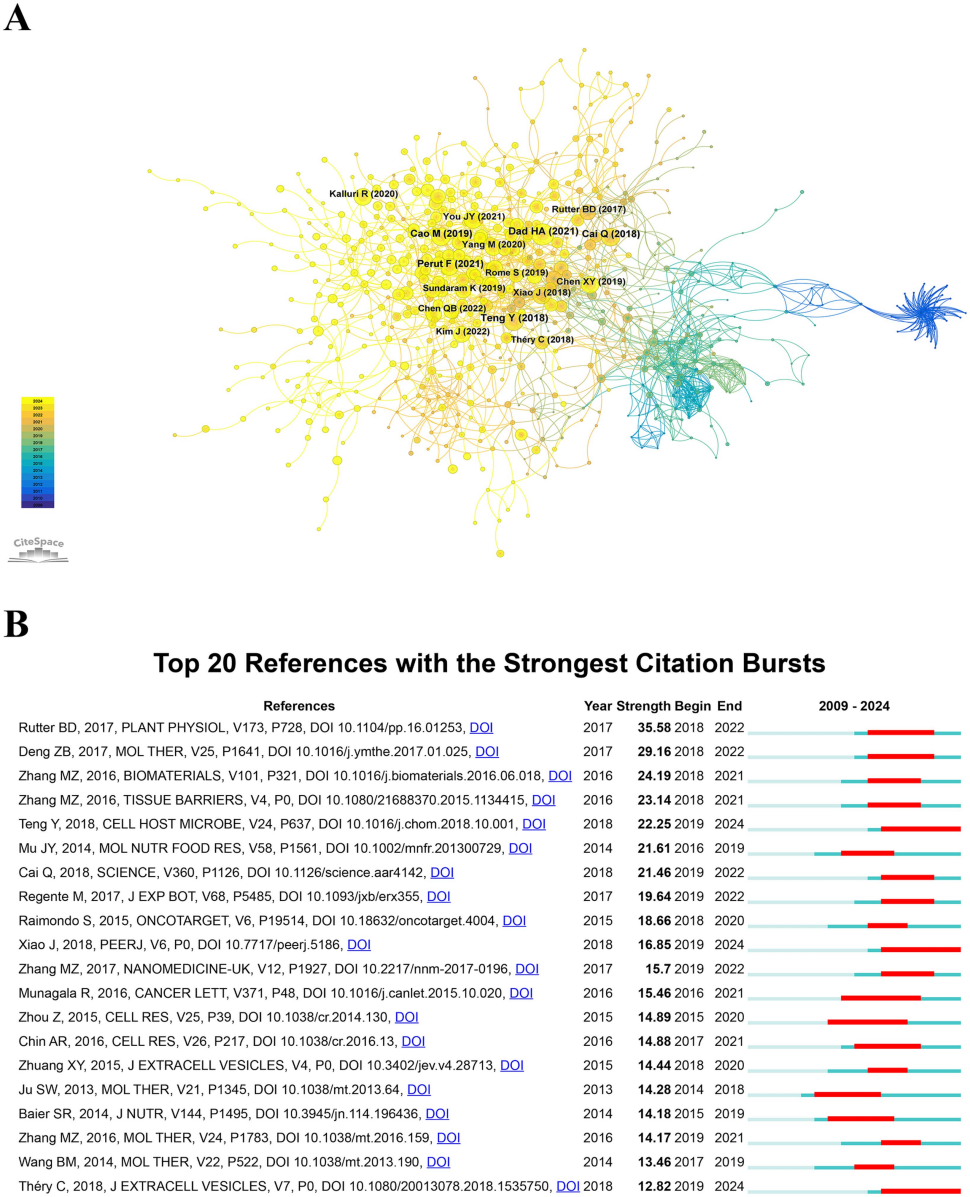
**(A)** A diagram portraying literature co-citation analysis. The combined dimensions of the overlapping spheres, each representing a yearly citation ring, are associated with the frequency of co-citations. Purple indicates earlier citations, while yellow represents more recent ones; blended colors signify citations spanning across these years. The lines connecting the spheres represent co-citation links, with prominent nodes highlighted in rose red. **(B)** A list of the Top 20 references distinguished by their notable citation bursts. A “burst” indicates a sudden increase in the significance of a research topic within a specified time period. “BurstBegin” marks the start of this rapid growth, while “BurstEnd” indicates the end of this growth phase.

Identifying highly cited and influential publications offers researchers a deeper comprehension of their field and assists in prioritizing future research directions. Table 1 presents the 10 most frequently cited publications concerning the theranostic applications of PDENs in healthcare, with eight of these works accumulating over 300 citations. The most referenced study, authored by Teng et al. (7) and published in *Cell Host & Microbe*, has been cited 674 times. Following this, the paper by Jingyao Mu et al. (2014), appearing in *Molecular Nutrition & Food Research*, has garnered 497 citations, while the study by Brian D. Rutter et al. (2017), published in *Plant Physiology*, has received 413 citations. Figure 7A illustrates a co-citation network of academic articles related to the theranostic uses of PDENs in healthcare, covering the period from January 1, 2009, to December 3, 2024, as analyzed using CiteSpace. The dimensions of each overlaid sphere, which represent the combined size of circles connected by tree ring lines, are directly proportional to the frequency of co-citations. Citations from earlier years are represented in blue, while more recent citations are depicted in yellow; the gradient of colors in between highlights the years during which the articles were cited. The lines connecting the circles illustrate the co-citation relationships among various works, and nodes highlighted in rose red mark the most influential references within this network. The top three publications with the highest co-citation frequencies are “Plant Exosome-like Nanovesicles: Emerging Therapeutics and Drug Delivery Nanoplatforms” (12), “Plant-Derived Exosomal MicroRNAs Shape the Gut Microbiota” (7), and “Plants send small RNAs in extracellular vesicles to fungal pathogen to silence virulence genes” (4), with co-citation frequencies of 163, 150, and 134, respectively. Notably, among the top 10 co-citation publications, the paper “Strawberry-Derived Exosome-Like Nanoparticles Prevent Oxidative Stress in Human Mesenchymal Stromal Cells” (59) stands out due to its high degree value, indicating that this node has more connections in the entire co-citation network and may play the role of a hub or bridge. The co-citation network analysis highlights the field’s foundational and emerging research trends, providing critical insights for stakeholders. Policymakers can utilize this information to fund impactful projects, researchers can build upon established knowledge to address current gaps and explore interdisciplinary opportunities, and industry practitioners can align their research and development strategies with influential research to maximize translational outcomes. The sustained relevance of these highly cited works emphasizes their significance in advancing the theranostic applications of PDENs in healthcare.

**Table 1:** Top 10 most highly cited literature in the field of the theranostic applications of plant-derived exosome nanovesicles in healthcare over the past 15 years.

Rank	Title	Type	Author (Year)	Journal	Citations (WoSCC)	DOI
1	Plant-Derived Exosomal MicroRNAs Shape the Gut Microbiota	Article	Teng et al. (7)	*Cell Host & Microbe*	674	10.1016/j.chom.2018.10.001
2	Interspecies communication between plant and mouse gut host cells through edible plant derived exosome-like nanoparticles	Article	Jingyao Mu et al. (110)	*Molecular Nutrition & Food Research*	497	10.1002/mnfr.201300729
3	Extracellular Vesicles Isolated from the Leaf Apoplast Carry Stress-Response Proteins	Article	Brian D Rutter et al. (111)	*Plant Physiology*	413	10.1104/pp.16.01253
4	Targeted Drug Delivery to Intestinal Macrophages by Bioactive Nanovesicles Released from Grapefruit	Article	Baomei Wang et al. (112)	*Molecular Therapy*	390	10.1038/mt.2013.190
5	*Citrus limon*-derived nanovesicles inhibit cancer cell proliferation and suppress CML xenograft growth by inducing TRAIL-mediated cell death	Article	Stefania Raimondo et al. (113)	*Oncotarget*	361	10.18632/oncotarget.4004
6	Plant Exosome-like Nanovesicles: Emerging Therapeutics and Drug Delivery Nanoplatforms	Review	Haseeb Anwar Dad et al. (12)	*Molecular Therapy*	342	10.1016/j.ymthe.2020.11.030
7	Ginseng-derived nanoparticles alter macrophage polarization to inhibit melanoma growth	Article	Meng Cao et al. (114)	*Journal for ImmunoTherapy of Cancer*	320	10.1186/s40425-019-0817-4
8	A review on exosomes application in clinical trials: perspective, questions, and challenges	Review	Jafar Rezaie et al. (115)	*Cell Communication and Signaling*	301	10.1186/s12964-022-00959-4
9	Pharmacological properties and derivatives of shikonin-A review in recent years	Review	Chuanjie Guo et al. (116)	*Pharmacological Research*	254	10.1016/j.phrs.2019.104463
10	Identification of exosome-like nanoparticle-derived microRNAs from 11 edible fruits and vegetables	Article	Juan Xiao et al. (63)	*PeerJ*	233	10.7717/peerj.5186

WoSCC, The Web of Science Core Collection.

The citation burst map of the top 20 most-cited publications related to the theranostics applications of PDENs in healthcare, spanning from January 1, 2009, to December 3, 2024, was analyzed via CiteSpace. A citation burst refers to a significant increase in the frequency at which a particular article is cited over a defined period. The red areas in the figure represent the intervals when the citation frequency of each publication rose sharply. As depicted in Figure 7B, the article by Brian D Rutter (2017) had the highest burst intensity (60), peaking at 35.58, with a citation surge occurring between 2018 and 2022. Following this, “Deng Zhongbin” (61) had a burst intensity of 29.16, with a burst period from 2018 to 2022. “Zhang Mingzhen” (62) ranks third in burst intensity, indicating that these literatures have considerable influence in the field of theranostics applications of PDENs in healthcare. Among the top 20 literatures, the citation burst periods of Teng Yun (7), Xiao Juan (63), and Théry Clotilde (64) are relatively late, all from 2019 to 2024, indicating that these studies have gained considerable attention and popularity in recent years and have become influential in the field of theranostics applications of PDENs in healthcare research. The citation burst analysis emphasizes the dynamic nature of research in theranostic applications of PDENs. Policymakers can allocate resources effectively to enhance high-impact areas, researchers can identify and address critical emerging trends, and industry practitioners can strategically align research and development efforts with influential studies. The identification of late-stage citation bursts highlights opportunities to explore recent, impactful research that continues to shape the future of PDENs-based healthcare solutions.

### 3.4 High-frequency keyword visualization

**Figure 8:**
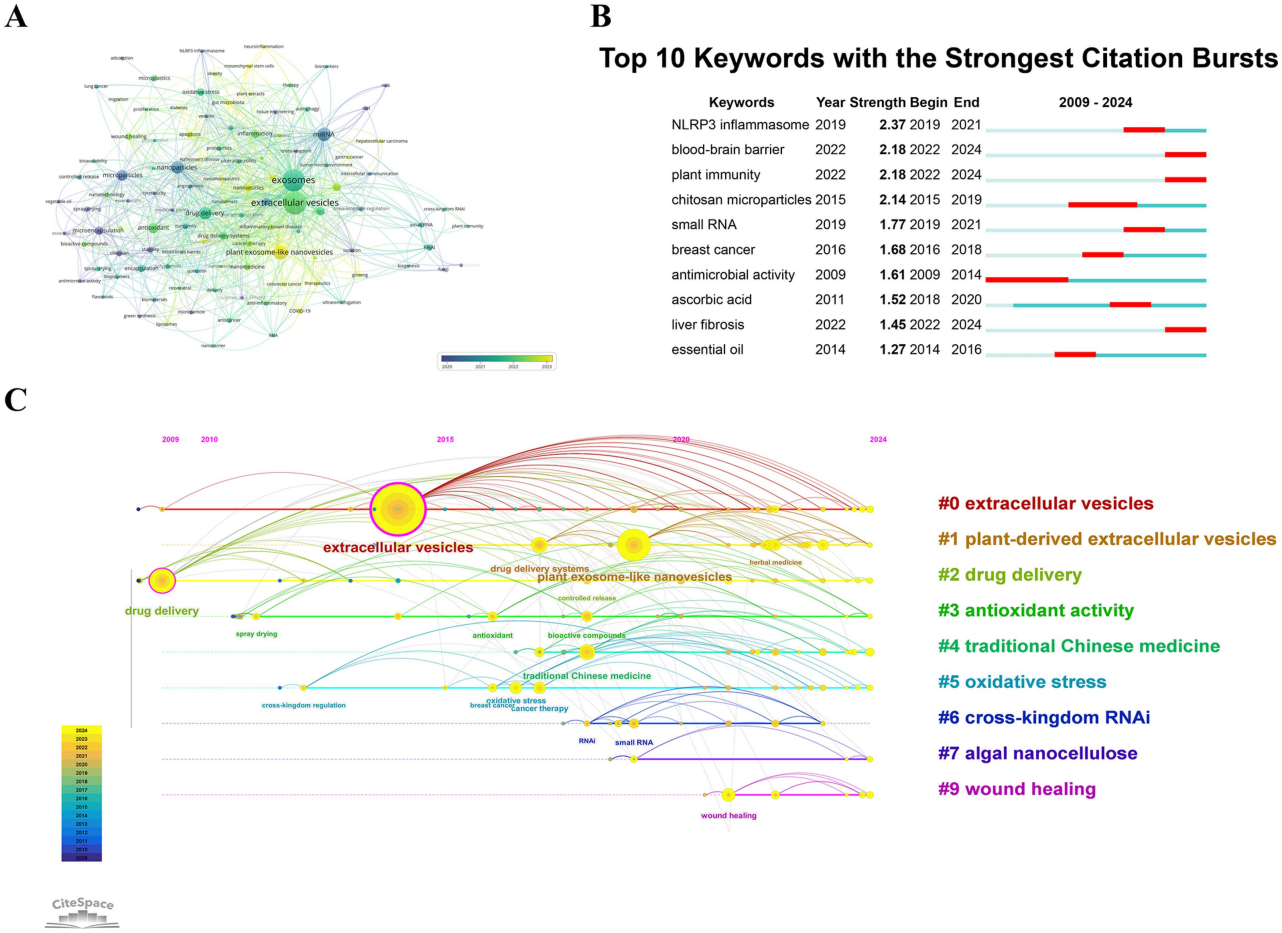
**(A)** A temporal representation of keyword intensity is depicted by circular nodes whose sizes reflect the frequency of keyword occurrences. The color gradient ranges from blue to yellow, highlighting the shift of keywords from earlier to more recent periods, thereby showcasing emerging research trends. **(B)** The top 10 keywords that exhibit the most significant citation bursts are determined by CiteSpace. A “burst” refers to a rapid increase in the prominence of a specific topic, with “BurstBegin” marking the onset and “BurstEnd” denoting the point at which this growth diminishes. **(C)** A timeline analysis that illustrates keyword clustering. The size of each sphere, corresponding to annual data, is proportional to the frequency of the keywords; the links between spheres indicate keyword co-occurrence. Purple indicates keywords that emerged earlier, yellow signifies those that appeared later, and mixed colors illustrate ongoing relevance. Key central nodes are highlighted in rose red. Keywords within the same cluster are aligned horizontally, with the earliest emergence times positioned at the top and progressing rightward. This arrangement facilitates the visualization of keyword volume and temporal distribution across clusters, illustrating their significance and duration.

VOSviewer performed a clustering analysis based on the co-occurrence of keywords, applying a minimum occurrence threshold of six. Keywords meeting this criterion were selected from a total of 4,487 distinct keywords and incorporated into the visualization. Figure 8A presents the keyword time atlas, where each node is represented by a circle along with its corresponding label. The size of each node correlates with the frequency at which a keyword appears. The color of each node reflects the average year of its occurrence, with a color gradient provided in the lower right corner. In this representation, blue signifies earlier years, while yellow denotes more recent ones. The analysis reveals that keywords like “chitosan” and “microencapsulation” exhibit a relatively earlier average occurrence, primarily before 2020. The average occurrence times of keywords like “exosomes,” “drug delivery,” and “nanoparticles” are between 2020 and 2022. The average occurrence times of keywords such as “plant exosome-like nanovesicles” and “traditional Chinese medicine” are from 2022 to 2024. These might be the research hotspots and directions in recent years. The chronological distribution of keywords offers valuable insights into the shifting research landscape of PDENs. The early prevalence of terms such as “chitosan” and “microencapsulation” indicates an initial focus on biomaterial-centered delivery approaches, particularly in enhancing stability and encapsulation efficiency. Between 2020 and 2022, the increasing prominence of keywords like “exosomes,” “drug delivery,” and “nanoparticles” underscores the expanding recognition of PDENs as bioactive carriers with superior biocompatibility. More recently, the emergence of phrases such as “plant exosome-like nanovesicles” and “traditional Chinese medicine” signals a transition toward harnessing plant-derived nanovesicles for therapeutic purposes, particularly within the realm of natural product-based medicine. This progression signifies a shift from foundational investigations of materials to translational applications, underscoring PDENs’ potential in personalized medicine, regenerative therapies, and precision-targeted drug delivery. The clustering analysis highlights evolving research trends, with keywords like “plant exosome-like nanovesicles” and “traditional Chinese medicine” emerging as recent hotspots. Policymakers, researchers, and industry practitioners can use these findings to align their priorities with emerging trends. By supporting research in recent hotspots and promoting collaboration across disciplines, stakeholders can speed up the translation of advanced PDENs-based technologies into effective healthcare solutions.

Figure 8B, analyzed by CiteSpace, shows the 10 keywords with the highest citation bursts in the study of “PDENs’ applications in biomedical research” from January 1, 2009, to December 3, 2024. This figure indicates a significant increase in the citation frequency of these keywords within a specific time frame. The red region in the figure shows when this citation surge occurred. It presents the burst phenomenon of citations for these keywords in terms of the year, intensity (Strength), start year (Begin), and end year (End), allowing for a quick assessment of the research trends and focal points in this domain in recent years. The keyword exhibiting the highest citation burst intensity is “NLRP3 inflammasome,” which recorded a burst intensity value of 2.37. This burst occurred over the period from 2019 to 2021. The keyword with the longest citation burst duration is “antimicrobial activity,” from 2009 to 2014, with a continuous growth for up to 6 years. Among the top 10 keywords with citation burst, those in recent years are “blood–brain barrier,” “plant immunity,” and “liver fibrosis,” suggesting that they might be the hotspots and directions of research in “PDENs’ applications in biomedical research” recently? The citation surges of these keywords illustrate the dynamic evolution of PDENs research by pinpointing emerging areas of interest. The increasing references to “blood–brain barrier” signal a growing focus on leveraging PDENs for drug delivery to the central nervous system, an area with substantial therapeutic potential yet formidable physiological barriers. Similarly, the heightened visibility of “plant immunity” reflects rising interest in the intrinsic role of PDENs in plant defense, which could inspire novel biomedical applications, particularly in immune-modulating therapies. The emergence of “liver fibrosis” suggests a widening scope of PDEN-related studies in hepatic disease models, marking a shift toward their application in chronic inflammatory conditions. These citation surges offer critical insight into shifting research priorities, underscoring the necessity for further experimental investigations, especially to refine PDENs’ functional attributes and elucidate their underlying mechanisms in disease modulation. Policymakers can prioritize funding and regulatory efforts in these emerging areas, especially those addressing critical health concerns such as neurological diseases and liver-related conditions, using this information. Supporting research in these hotspots may have a lasting impact on healthcare policy and patient outcomes. Industry professionals can guide the development of new products and treatments by leveraging these emerging trends. Additionally, the prolonged interest in “antimicrobial activity” implies ongoing market demand for therapeutic solutions in this area. By aligning funding, research agendas, and product development efforts with these emerging trends, stakeholders can accelerate advancements in the field and address critical health challenges more effectively. Beyond influencing research priorities, the persistent citation surges of keywords such as “antimicrobial activity” reflect enduring scientific and commercial enthusiasm for PDEN applications. The sustained prominence of this term suggests a consistent demand for PDEN-based antimicrobial and anti-inflammatory therapies, particularly in the context of combating antibiotic resistance. This ongoing trend highlights the necessity for more in-depth mechanistic investigations and translational research aimed at optimizing PDENs for clinical applications in infectious disease treatment.

Examining the trends and key areas within the “applications of PDENs in biomedical research” is essential for informing funding decisions and facilitating collaborations, aligning them with current research priorities. Figure 8C is the timeline analysis after clustering the keywords related to “PDENs’ applications in biomedical research.” The size of the superimposed spheres, that is, the sum of the sizes of the spheres corresponding to the tree ring lines, is proportional to the keyword frequency. The lines connecting the keywords indicate instances of co-occurrence. Purple represents keywords that appeared earlier, and yellow indicates those that emerged more recently. Overlapping colors highlight the specific years when these keywords were active. The nodes marked in rose red signify those with higher centrality, indicating their crucial role and positioning within the network. Keywords of the same cluster are placed on the same horizontal line. The time of the first appearance of the keywords is at the top of the view, and the time gets closer as it moves to the right. Through this figure, one can obtain the number of keywords in each cluster. The more keywords there are, the more significant the cluster field is. The temporal distribution of keywords within each cluster can also be determined. As shown in the figure, the keywords are organized into 10 distinct clusters: #0 extracellular vesicles, #1 plant-derived extracellular vesicles, #2 drug delivery, #3 antioxidant activity, #4 traditional Chinese medicine, #5 oxidative stress, #6 cross-kingdom RNAi, #7 algal nanocellulose, #9 wound healing. Clustering continues to evolve in the realm of “PDENs’ applications in biomedical research.” These findings can guide policymaking by highlighting which research topics should be prioritized for funding, focusing on biomedical applications of PDENs that can have broad health and economic benefits. The clustering analysis delineates distinct thematic domains within PDENs research, each signifying an area of active exploration. Established clusters, including “extracellular vesicles,” “oxidative stress,” and “drug delivery,” emphasize PDENs’ well-recognized biomedical significance, whereas emerging clusters such as “cross-kingdom RNAi” and “wound healing” reveal novel therapeutic prospects. The presence of a cluster centered on “traditional Chinese medicine” suggests a growing convergence between plant-derived nanovesicles and herbal medicine, pointing to their potential as bioactive cargo delivery systems. These thematic groupings not only encapsulate prevailing research trajectories but also provide strategic insights for future investigations, particularly in refining PDENs for enhanced therapeutic effectiveness and advancing scalable manufacturing approaches for clinical implementation. Policymakers can use this data to strategically allocate resources to high-impact areas such as wound healing, antioxidant activity, and cross-kingdom RNAi. By focusing on these trending topics, researchers can align their studies with the broader scientific discourse, ensuring their work remains at the cutting edge of innovation. Additionally, the identification of central concepts can direct researchers toward key areas with high collaborative potential. Industry professionals can leverage these findings to identify the most promising areas for technological development, product innovation, and commercialization.

### 3.5 Related diseases

**Figure 9:**
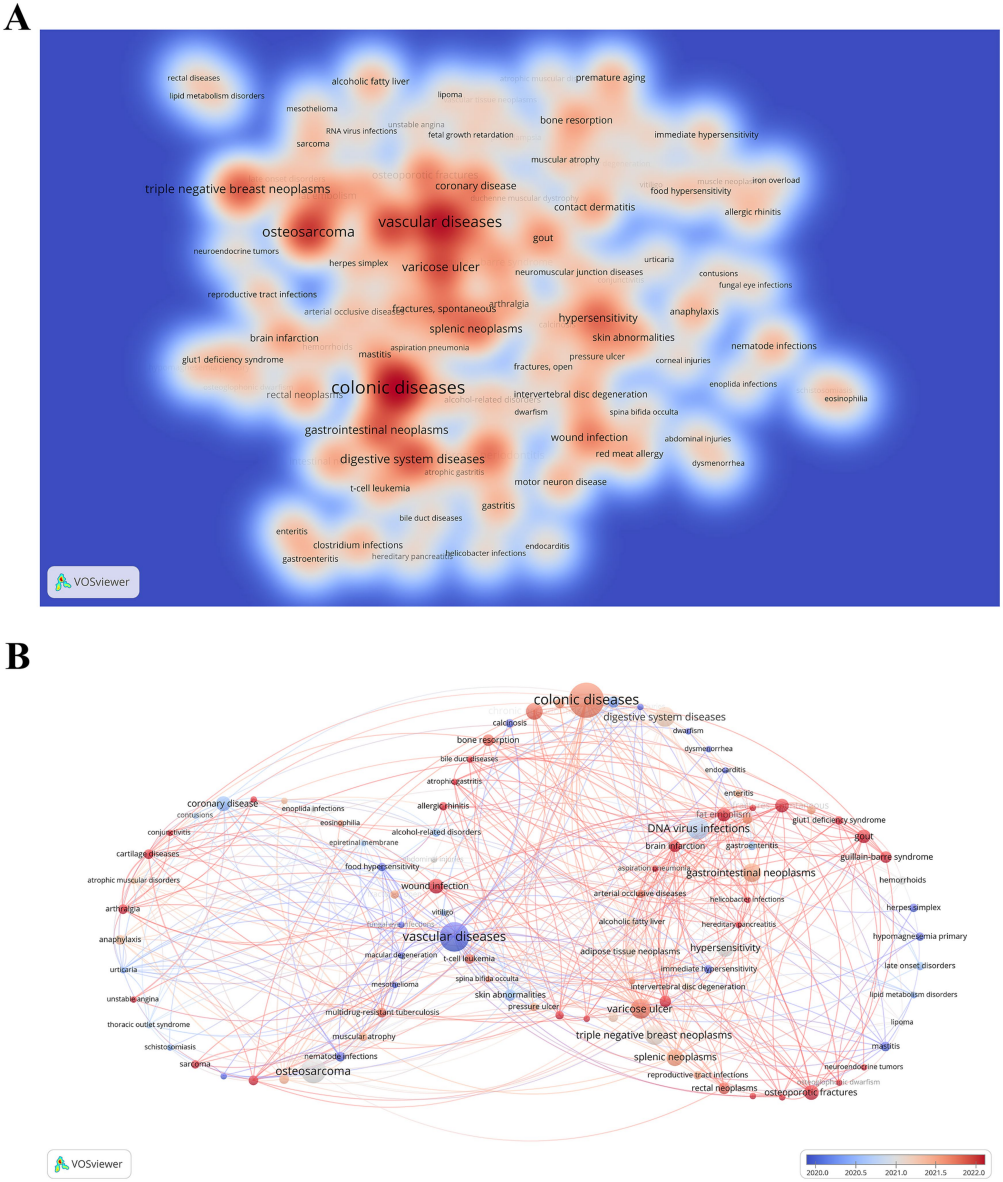
**(A)** A density visualization map depicting related diseases, with variations in color intensity indicating the frequency of occurrence of each disease. **(B)** Time atlas of disease occurrence. In the figure, a circle and a label constitute a node. The size of the circle is positively correlated with the frequency of the disease occurrence. The color of each sphere is represented by the color gradient in the lower right corner, indicating the average occurrence year. Blue represents that the occurrence time of the disease is relatively early; red represents that the occurrence time of the disease is relatively late.

**Figure 10:**
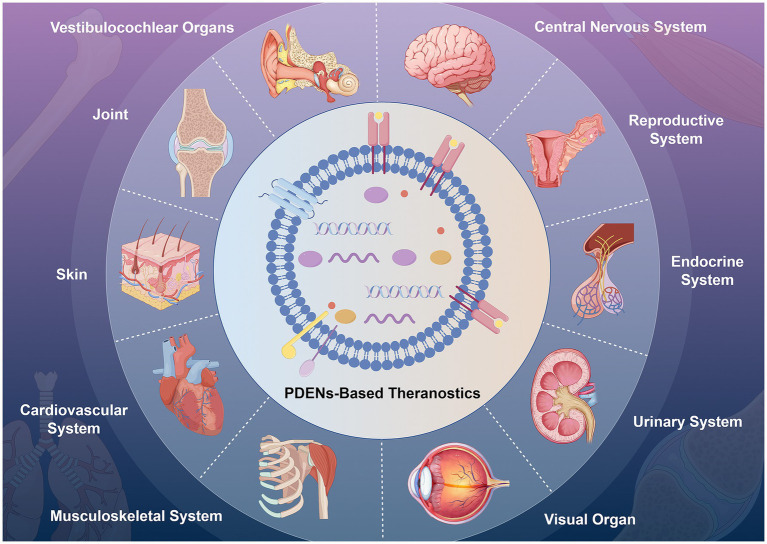
Future studies should explore the broader application of plant-derived exosome nanovesicles in theranostics across a wider range of diseases. This figure was created using Figdraw (https://www.figdraw.com/static/index.html#/). PDENs, plant-derived exosome nanovesicles.

By identifying the diseases most related to PDENs’ applications in biomedical research, scientists can focus on specific health issues and potentially accelerate the development of targeted pharmaceuticals and therapies. The Citexs Data Platform has listed 723 distinct diseases. These were mentioned in 1,549 academic articles, and each disease was cited in at least three publications. VOSviewer produced a heatmap that visualizes the prevalence and interrelations of these diseases within this field (Figure 9A). The four most prevalent conditions identified are colonic diseases, vascular diseases, osteosarcoma, and DNA virus infections. Additionally, VOSviewer conducted a time atlas analysis of these diseases based on co-occurrence (Figure 9B). In this graphical representation, a circle and a label form a node. The size of the circle is positively correlated with the frequency of the disease’s occurrence. The color of each sphere is indicated by the color gradient in the lower right corner, representing the average occurrence year. Blue means the occurrence time of the disease is relatively early; red means it is relatively late. The figure shows that the average occurrence time of vascular diseases is relatively early, from 2019 to 2020. The average occurrence time of colonic diseases and osteosarcoma is relatively late, from 2021 to 2022, and they may be the hot diseases in this field in recent years. Based on the outcomes of the above two figures, we can easily discover that the main focus of current research is on diseases of the gastrointestinal and circulatory systems, as well as malignant tumors. However, significant knowledge gaps remain in exploring the potential of PDENs for disorders of the hematological, endocrine, nervous, and degenerative musculoskeletal systems. This observation aligns with earlier findings in the literature (1, 25, 38). Expanding research on the therapeutic potential of PDENs for conditions in these underexplored areas could lead to identifying additional clinical applications (Figure 10). Policymakers can use these insights to allocate funding effectively. Researchers can identify emerging trends and fill existing knowledge gaps. Industry practitioners can use these findings to guide product development and target emerging markets, ensuring that the therapeutic potential of PDENs is fully realized across a wide range of diseases.

### 3.6 Key challenges and outlook

Since their discovery, PDENs have shown exceptional potential in biological therapy, drug delivery, and overcoming biological barriers, establishing them as a promising focus in the biomedical field (27). PDENs, as a cell-free therapeutic approach, offer key advantages such as biocompatibility, low toxicity, environmental sustainability, stability, cost-effectiveness, and intrinsic therapeutic properties (25, 65). PDENs function as both innovative nanocarriers and eco-friendly nano-factories, utilizing their biological features and regulatory modifier sites for efficient drug delivery (66). Moreover, PDENs can be mass-produced from abundant plant-derived resources, unlocking new possibilities in clinical and biomedical applications (29). As a result, various eco-friendly PDENs have opened up new possibilities for advancing clinical and biomedical applications.

**Figure 11:**
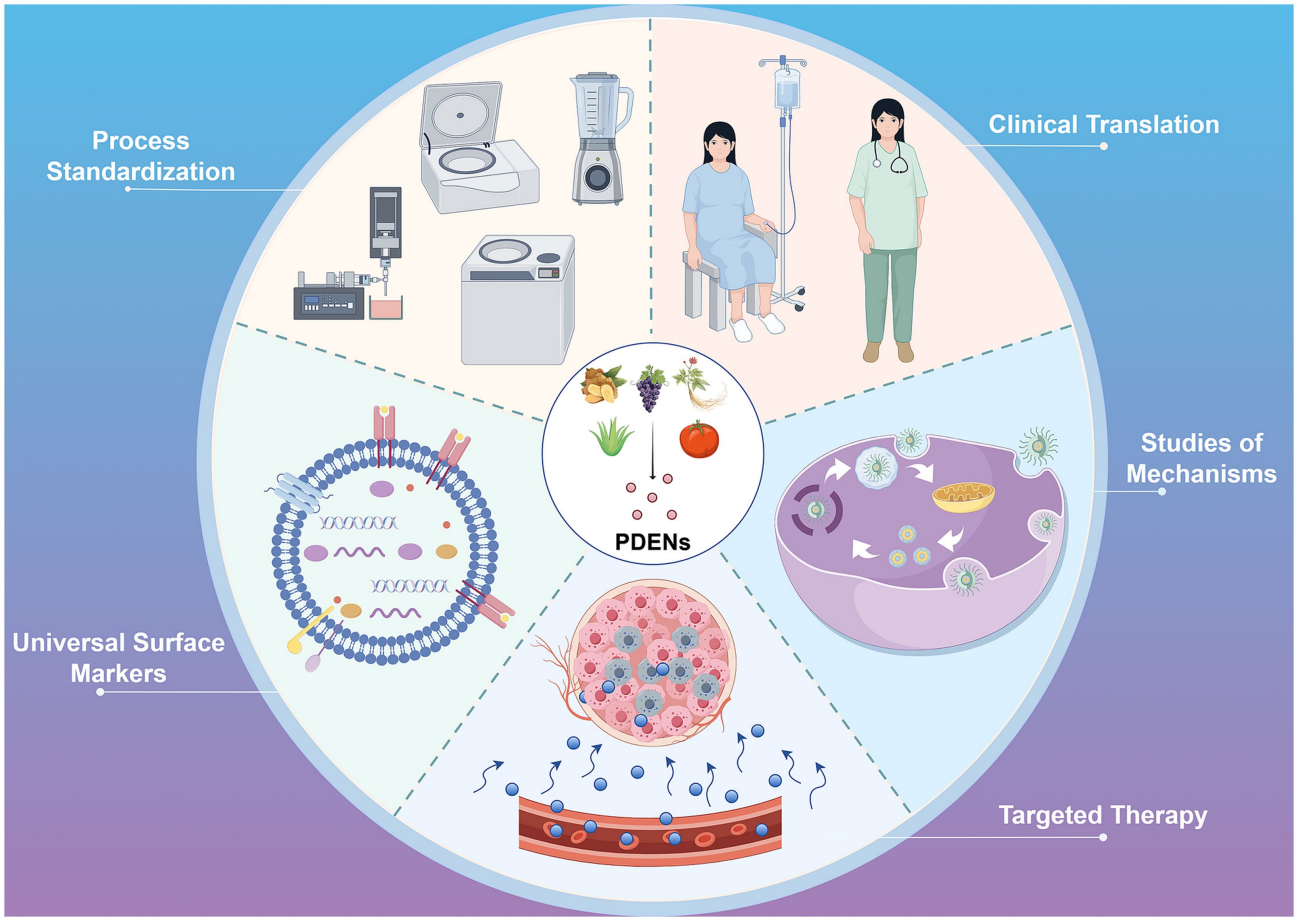
Key challenges in current research on plant-derived exosome nanovesicles. This figure was created using Figdraw (https://www.figdraw.com/static/index.html#/). PDENs, plant-derived exosome nanovesicles.

Despite progress, PDENs research remains underdeveloped. Bridging the gap between laboratory studies and clinical-scale production requires further in-depth investigation and collaborative efforts. Key challenges that must be addressed include the following (Figure 11):

1. Nomenclature Issues: The lack of standardized nomenclature for PDENs is a significant issue (1, 67, 68). Due to their distinct mechanisms and characteristics compared to animal-derived exosomes, a separate and clear naming system is essential. Recent consensus on naming extracellular vesicle-like particles from traditional Chinese medicine provides valuable guidance for researchers globally (69).
2. Extraction, Purification & Characterization Challenges: Extracting and purifying PDENs with high purity and yield is challenging due to plant cell walls, requiring expensive equipment and complex protocols. Current methods do not meet the demands of clinical-scale production (70). Currently, no universal protocols exist for PDENs characterization or quality control, both critical for consistent and efficient production (71, 72). Variations in PDENs size, morphology, and active compound concentrations can arise based on plant species, plant parts, and environmental or seasonal factors. Such variability contributes to inconsistencies in the extracted material (30). Such heterogeneity hampers experimental reproducibility, production scalability, and regulatory approval across jurisdictions (73). Consequently, we suggest the creation of a new task force focused on the standardization of PDENs by the International Society of Extracellular Vesicles. This is to ensure the safe and effective use of PDENs in therapeutic applications (74). The incorporation of artificial intelligence (AI) and machine learning (ML) into the field of nanomedicine holds significant potential for enhancing the processes of PDENs extraction and purification. These technologies can optimize conditions, enhance scalability, and maintain strict quality control standards (75, 76). Given the variable nature of plant sources, a universal method might not be feasible (21). We propose developing general principles tailored to plant-specific characteristics to create a framework for diversifying plant sources.
3. Insufficient In-depth Mechanism Research on PDENs: Although PDENs have significant therapeutic potential, the mechanisms underlying their biological activity—such as cellular uptake, cargo delivery, and intracellular trafficking—are not yet fully understood (36, 77). This gap in knowledge may limit their clinical use. An incomplete understanding of how PDENs exert their effects can hinder their design and optimization for specific applications. For instance, in drug delivery, unpredictable release patterns and interactions with biological membranes can lead to suboptimal therapeutic outcomes (11). Advanced approaches, including deep learning and systems biology, provide valuable tools for modeling the interactions between PDENs and target cells, helping predict their therapeutic efficacy and inform the design of more effective exosome-based treatments (78, 79). Additionally, integrating multi-omics techniques—such as transcriptomics, proteomics, lipidomics, and metabolomics—can provide comprehensive profiles of PDENs, offering critical insights into their cargo composition and the mechanisms of their therapeutic effects (80–82).
4. Absence of Universal Surface Markers for PDENs: The diversity of plant sources that produce PDENs leads to the lack of universally recognized surface markers, hindering standardization in identification (83, 84). In 2021, Marcela Pinedo and colleagues identified three common protein families within PDENs and proposed three candidate marker proteins: heat shock protein 70, S-adenosyl-homocysteinase, and glyceraldehyde 3-phosphate dehydrogenase (85). However, these suggestions have not been universally accepted. Therefore, further proteomic studies on PDENs are needed to identify a set of conserved, reliable, and broadly recognized markers. Currently, no commercially available antibodies target these proposed markers.
5. From Bench to Bedside: Current research on PDENs for theranostic purposes primarily involves *in vitro* cell assays and *in vivo* rodent models. Although PDENs are commonly derived from edible plants like fruits, vegetables, and herbs, the metabolic pathways for their degradation and elimination are poorly understood. This raises concerns about potential bioaccumulation and toxicity (86). Further research is needed to ensure the safe utilization of PDENs in nanomedicine and tissue regeneration prior to their clinical application. Key aspects to address include long-term pharmacokinetic and pharmacodynamic profiles, biodistribution, immune response, and potential toxicity (30, 87, 88). Moreover, comprehensive studies and follow-up are essential to assess the therapeutic efficacy and potential adverse effects of PDENs. ML algorithms can predict the toxicity, biodistribution, and immunogenicity of PDENs based on preclinical data, expediting safety assessments and aiding regulatory approval (89, 90). *In vitro* three-dimensional models, like organoids that mimic human tissues, offer a more physiologically relevant platform for PDENs research. These models enhance predictive accuracy for clinical outcomes, reduce reliance on animal testing, and facilitate translation into clinical applications (91–94).
6. Enhanced Targeted Therapy for PDENs is required: in precision medicine, functionalizing PDENs for targeted delivery to specific tissues or cells is a significant challenge (41). Low targeting efficiency can lead to unintended effects, reducing the therapeutic effectiveness of PDENs. Currently, PDENs distribution in both *in vitro* and *in vivo* studies depends largely on their natural properties, without active targeting strategies (95). Therefore, medicinal chemists, pharmacists, and pharmacologists must explore strategies for engineering PDENs—such as surface modifications and genetic engineering techniques—to improve targeting efficiency and ensure accurate drug delivery (96, 97). AI can help design and optimize surface modifications, predicting the most effective ligands for targeted drug delivery (75). AI can also analyze patient-specific data, providing a valuable tool for developing personalized medicine strategies with PDENs (98, 99).
7. Investigation into Novel Administration Modes of PDENs: Most animal studies on PDENs have focused on oral administration, with fewer exploring intravenous injection as an alternative (25, 38). Oral administration offers simplicity, convenience, and relative safety. However, first-pass elimination in the liver significantly reduces the drug’s concentration and dose in circulation (100). In contrast, intravenous administration provides faster, more durable effects but carries a higher risk of adverse reactions (101). Therefore, investigating additional administration routes, such as local injection and topical application, is crucial to determine the most effective delivery strategies for various diseases.
8. Stability of PDENs for Long-Term Storage: The state of the plants and the storage conditions of PDENs can influence their biological activities (81). PDENs from plant sources are prone to aggregation and degradation over time, which reduces their efficacy and shortens their shelf life (11). Environmental factors such as temperature, light, and humidity can worsen these stability issues, necessitating sophisticated and costly storage solutions to maintain their integrity over time (35). Therefore, more detailed studies on long-term stability are needed.
9. Trade-off of PDENs in Theranostics and Food Security: The growing global population and climate change create urgent demands for higher agricultural productivity (102). In this context, using PDENs for theranostics instead of as direct food sources could raise food security concerns (103). Allocating resources like arable land and water for theranostics could reduce food production, leading to higher food prices and increased scarcity, particularly in developing regions and fragile environments. Therefore, the trade-off between using plant-derived materials for biomedical applications and maintaining food security must be considered. A potential solution is to explore biomaterials derived from plant waste or by-products (104).

Despite ongoing challenges, PDENs show great potential for theranostic applications due to their biocompatibility, bioactivity, and natural abundance. Addressing these barriers through technological advancements and interdisciplinary research can lead to more efficient, clinically applicable solutions. This will position PDENs as a key component in the future of healthcare and expand our understanding of the field.

## 4 Strengths and limitations

Unlike previous studies relying on meta-analyses or narrative reviews, this research employs bibliometric techniques for a more detailed examination of research trends and key focus areas (105). This study uses bibliometric analysis to explore the theranostic potential of PDENs, making it one of the first efforts to map the knowledge landscape of this emerging field. Despite certain limitations, this study provides a robust foundation and critical insights to guide future research. These findings are expected to advance PDENs-related research.

However, this study has several limitations. First, using CiteSpace for data retrieval limited the dataset to publications in the WoSCC database, potentially introducing selection bias (106). The selection of a bibliometric database plays a crucial role in shaping research outcomes, as variations in journal coverage, document classifications, and disciplinary focus can lead to disparities in retrieved data. For example, while Scopus encompasses a wider range of engineering and regionally focused journals, PubMed is dedicated exclusively to biomedical literature. These discrepancies may result in the inadvertent exclusion of pertinent studies within this field of inquiry. To achieve a more exhaustive evaluation of research trajectories and significant developments in the theranostic applications of PDENs in healthcare, future investigations should incorporate data from multiple databases, thereby mitigating potential biases associated with reliance on a single source. Second, relying solely on citation counts to assess a paper’s impact may be imprecise due to external influences (107). Third, the large volume of literature limited detailed investigations of individual studies and subfields, potentially reducing the depth of findings. Fourth, the use of natural language processing techniques in bibliometric analyses may introduce biases in data interpretation, as noted in prior research (56, 105, 108). Fifth, restricting this study to English-language publications may cause publication bias (109), though the global use of English in science lessens its impact. Future research should include multilingual databases for a broader understanding of the field. Sources like the China National Knowledge Infrastructure, SinoMed, Wanfang Database, and Weipu Database are recommended for analyzing trends in non-English and regional studies. Sixth, the partial retrieval of pertinent literature may have resulted in the exclusion of recent publications and the omission of emerging terminology. Finally, a notable constraint of current bibliometric methodologies lies in their incapacity to directly capture data pertaining to translational outcomes, including patents, clinical trials, and commercial applications. Predominantly, academic repositories such as WoSCC emphasize peer-reviewed literature, encompassing articles and reviews, whereas patent databases (e.g., the United States Patent and Trademark Office) and clinical trial registries (e.g., ClinicalTrials.gov) operate within distinct data infrastructures. While our investigation identified 1,549 scholarly works related to the theranostic applications of PDENs in healthcare from 2009 to 2024, the extent to which these contributions resulted in patent filings or clinical trials remains indeterminate. This limitation stems from the fact that such data is primarily housed within industry reports and regulatory filings, which are not routinely included in academic journal databases. Addressing this issue necessitates the incorporation of patent and clinical trial data into future bibliometric research. Although this integration presents challenges—such as disparities in data structuring and access limitations—it holds the potential to offer a more holistic perspective on the progression from fundamental research to practical implementation.

## 5 Conclusion

Considerable advancements have been achieved in the domain of PDENs over the past 15 years. Integrating the unique properties of PDENs with biomedical systems could accelerate their transition from laboratory research to clinical practice by maximizing benefits and minimizing drawbacks. This study addresses this transition by analyzing advancements and challenges in the theranostic applications of PDENs in healthcare. Using visual analysis, we evaluate the contributions and collaborations of countries, institutions, and researchers to provide a comprehensive view of the research landscape. We further analyze journal distributions and co-citation networks to assess research impact and future trends. Additionally, we analyze co-cited references, citation bursts, and keywords to identify emerging research areas and future trajectories. As research advances, PDENs are expected to play a crucial role in developing innovative biomedical technologies for diagnosing and treating diseases. In summary, this research presents a comprehensive visualization of the PDENs landscape, shedding light on the current research trends and exploring potential avenues for future investigation. We hope this study will encourage further exploration of PDENs applications in healthcare.

## Data Availability

The raw data supporting the conclusions of this article will be made available by the authors, without undue reservation.

## Footnotes

^1^ https://www.graphica.app/

^2^ https://www.citexs.com
