# Supplementary material for: Unveiling the dynamic trends of plant-derived exosome nanovesicles-based theranostics: through bibliometric and visualized analysis
Source: Front Med (Lausanne). 2025 Jun 24;12:1553915. doi: 10.3389/fmed.2025.1553915 (PMC12234478; doi:10.3389/fmed.2025.1553915)
Supplement: Supplementary file 1 [file Table_1.docx]

Supplementary Materials

# Search strategy

| **Search** | **Query** |
| --- | --- |
| #1 | TS=(“plant” OR “herb” OR “plants” or “herbal” or “botanical” OR “botanic” OR “vegetal” OR “vegetable” OR “vegetables” OR “fruit” OR “fruits” OR “floristic” OR “floral” OR “foliage” or “vegetation” OR “garden stuff” OR “greenstuff” OR “greens” OR “fruitage” OR “herbaceous” OR “herbage” or “Traditional Chinese Medicine” OR “Phyto*”) |
| #2 | TS=(“exosomes” OR “exosome” OR “extracellular vesicle” OR “vesicle, extracellular” OR “vesicles, extracellular” OR “exovesicles” OR “exovesicle” OR “extracellular vesicles” OR “microvesicles” OR “microparticles” OR “ectosomes” OR “oncosomes” OR “nanovesicle” OR “Nanovesicles” OR “vesiclelike nanoparticles” OR “vesiclelike nanoparticle” OR “exosome nanovesicles” OR “exosomes-like nanoparticles” OR “exosomes-like nanovesicles” OR “exosome‑like nanocarriers” OR “exosome-like nanoparticles” OR “vesicle-like nanoparticles” OR “edible nanoparticles” OR “decoctosome” OR “botanosome”) |
| #3 | TS=(“pharmac*” OR “biomaterial*” OR “medic*” OR “biomedic*” OR “nanomedic*” OR “cancer” OR “neoplasms*” OR “disease*” OR “diagnos*” OR “bioimaging” OR “*therap*” OR “theranos*” OR “drug deliver*” OR “tissue engineer*” OR “healthcare” OR “biodevice*” OR“medical device*” OR “biolog*” OR “therapeutics” OR “therapeutic” OR “therapies” OR “treatment” OR “treatments” OR “biomedical*” OR “antibacterial*” OR “anti-inflammatory” OR “biosensor*” OR “diagnostic imaging*” OR “antiviral*” OR “anticancer*” OR “regeneration*” OR “immunoregulation*”) |
| #4 | #1 AND #2 AND #3 |
